# STAT3 signaling modulates the immune response induced after antigen targeting to conventional type 1 dendritic cells through the DEC205 receptor

**DOI:** 10.3389/fimmu.2022.1006996

**Published:** 2022-10-18

**Authors:** Fernando Bandeira Sulczewski, Larissa Alves Martino, Davi Salles, Márcio Massao Yamamoto, Daniela Santoro Rosa, Silvia Beatriz Boscardin

**Affiliations:** ^1^ Departamento de Parasitologia, Instituto de Ciencias Biomedicas, Universidade de Sao Paulo, Sao Paulo, Brazil; ^2^ Departamento de Microbiologia, Imunologia e Parasitologia, Universidade Federal de Sao Paulo, Sao Paulo, Brazil; ^3^ Instituto de Investigação em Imunologia (iii), INCT, Sao Paulo, Brazil

**Keywords:** dendritic cells, STAT3, maturation, T follicular helper cells, Th1, JAK/STAT signaling pathway

## Abstract

Conventional dendritic cells (cDC) are a group of antigen-presenting cells specialized in priming T cell responses. In mice, splenic cDC are divided into conventional type 1 DC (cDC1) and conventional type 2 (cDC2). cDC1 are specialized to prime the Th1 CD4^+^ T cell response, while cDC2 are mainly associated with the induction of follicular helper T cell responses to support germinal center formation. However, the mechanisms that control the functions of cDC1 and cDC2 are not fully understood, especially the signaling pathways that can modulate their ability to promote different CD4^+^ T cell responses. Here, we targeted a model antigen for cDC1 and cDC2, through DEC205 and DCIR2 receptors, respectively, to study the role of the STAT3 signaling pathway in the ability of these cells to prime CD4^+^ T cells. Our results show that, in the absence of the STAT3 signaling pathway, antigen targeting to cDC2 induced similar frequencies of Tfh cells between STAT3-deficient mice compared to fully competent mice. On the other hand, Th1 and Th1-like Tfh cell responses were significantly reduced in STAT3-deficient mice after antigen targeting to cDC1 *via* the DEC205 receptor. In summary, our results indicate that STAT3 signaling does not control the ability of cDC2 to promote Tfh cell responses after antigen targeting *via* the DCIR2 receptor, but modulates the function of cDC1 to promote Th1 and Th1-like Tfh T cell responses after antigen targeting *via* the DEC205 receptor.

## Introduction

Conventional dendritic cells (cDC) are bone marrow-derived antigen presenting cells responsible to initiate T cell responses to a myriad of different types of pathogens (1). Similarly, cDC are able to control tolerogenic immune responses to self-antigens to prevent the establishment of autoimmune diseases (2). To distinguish between immunogenic and tolerogenic stimuli, cDC express pattern recognition receptors (PPRs) and endocytic receptors that are able to sense pathogens, tissue damage and uptake their antigens (3). When cDC recognize antigens, they increase the expression of costimulatory molecules, MHCII and cytokines in order to present antigens to T cells and efficiently prime antigen-specific immune responses (4, 5).

cDC are classified into two major subsets: conventional type 1 dendritic cells (cDC1) and conventional type 2 dendritic cells (cDC2) (6). cDC1 and cDC2 differ based on their ontogeny, expression of membrane markers, localization in lymphoid tissues, and functions (7). In mice, splenic cDC1 are located in the red pulp (migratory cDC1) and in the white pulp (resident cDC1), more specifically in the center of the T cell zone of the spleen, and are specialized in antigen cross presentation (7–10). On the contrary, murine splenic cDC2 are localized in the bridging channels and in the marginal zone of B and T cell zones, or in the outer part of the T cell zone (8, 11). cDC2 are mainly associated with their ability to present antigens in the context of MHCII (9).

Much has been done to identify the unique roles of cDC1 and cDC2, specially on their differential ability to prime different CD4^+^ T cell responses (12–26). An efficient strategy to assess the function and biology of cDC *in vivo* consists of delivering antigens directly to these cells using chimeric monoclonal antibodies (mAb). The αDEC205 mAb has been widely used to target antigens to cDC1 *via* the DEC205 receptor. Antigen targeting to cDC1 promotes Th1 and Th1-like Tfh CD4^+^ T cell responses after immunization (8, 11, 13–15, 17–29). The αDCIR2 (33D1) mAb has been used to target antigens to cDC2. Mice immunization with αDCIR2 fused with antigens induces Tfh cell priming, suggesting that cDC2 are specialized to promote Tfh responses (21, 22, 24). Nonetheless, the mechanisms that control the function of cDC1 and cDC2 are still poorly understood.

Since cDC1 and cDC2 are differentially localized in lymphoid tissues, we asked whether different cytokines could specifically modulate their function. Signal Transducer and Activator of Transcription proteins (STAT) are a group of signal transducer proteins that are activated after a cytokine or a growth factor specifically recognize their receptors (30). STAT proteins participate in signaling pathways downstream to proteins known as JAK (Janus Associated Kinase) or Tyk2 (Tyrosine Kinase 2) (31). For example, STAT3 is activated through signaling of the cytokines IL-6, IL-10, IL-11, IL-21 and the growth factor GM-CSF (32). In DC, STAT3 signaling was firstly associated with the induction of a tolerogenic immune response due to its role in downregulation of costimulatory molecules (33–38). However, there is also evidence indicating that the STAT3 signaling pathway is associated with cDC1 maturation after stimulation with Poly (I:C), a TLR3 ligand, which promotes cDC1 maturation *via* type I interferon pathway (19, 39). In this way, the STAT3 signaling pathway may also be involved in the stimulation of cDC1 to induce inflammatory responses (39).

In an attempt to better understand the role of the STAT3 signaling pathway in cDC, we took advantage of CD11c^cre^STAT3^Flox/Flox^ (STAT3 cKO) mice to analyze to effect of the ablation of STAT3 signaling specifically on these cells. We used a previously characterized model of antigen targeting to cDC1 *via* DEC205 receptor and to cDC2 *via* DCIR2 receptor, using Poly (I:C) as an adjuvant (24). We targeted a model antigen composed by a fragment of 19 kDa from the merozoite surface protein 1 (MSP1_19_) derived from *Plasmodium vivax* conjugated to the synthetic pan allelic DR epitope (PADRE) (13, 40). Our findings indicate that cDC2 promote Tfh cell responses in a STAT3-independent manner, while STAT3 signaling stimulates cDC1 to promote Th1 and Th1-like Tfh cell responses after antigen targeting.

## Materials and methods

### Mice

Male and female STAT3 cKO, STAT3^Flox/Flox^ and C57BL/6 (WT) mice aged between 5-7 weeks were used. The CD11c^Cre^STAT3^Flox/Flox^ (STAT3 cKO) mouse strain was obtained by crossing CD11c-Cre (B6.Cg-Tg(Itgax-cre)1-1Reiz) (41) with STAT3^Flox/Flox^ (B6.129S1-Stat3^tm1Xyfu^) (42) mice purchased from The Jackson Laboratory (JAX stock #008068 and JAX stock #016923, respectively). Two breeding steps were performed: first, CD11c^Cre^ mice were crossed with STAT3^Flox/Flox^ and the offspring were genotyped. CD11c^Cre^-STAT3^Flox/WT^ F1 were selected and again crossed with STAT3^Flox/Flox^. The F2 mice were genotyped, and individuals with the CD11c^Cre^STAT3^Flox/Flox^ (referred as STAT3 cKO from now on) genotype were selected. STAT3 ^Flox/Flox^ animals from F2 were used as controls in the experiments. Mice were bred and maintained in the Animal Facility of the Department of Parasitology of the Institute of Biomedical Sciences of the University of São Paulo. The animals were kept under pathogen-free conditions, with water and food *ad libitum*. This study was approved by The Institutional Animal Care and Use Committee (IACUC) of the Institute of Biomedical Sciences of the University of São Paulo under the protocol number 7937100118, in agreement with the Brazilian national law on animal care (11.794/2008) and the ARRIVE guideline.

### Immunizations, spleen cells and blood samples

Depending on the experiment, STAT3 cKO, STAT3^Flox/Flox^ and WT mice were immunized intraperitoneally with 5 µg of αDEC205-MSP1_19_PADRE or αDCIR2-MSP1_19_PADRE mAbs together with 50 µg of Polyinosinic:polycytidylic acid [Poly (I:C)] (Invivogen) as adjuvant or only 50 µg Poly (I:C), as a negative control. In the indicated experiments, a second dose of immunization with 5 µg of the αDEC205-MSP1_19_PADRE mAb was also administered without the use of adjuvant 14 days after the first dose. The chimeric mAbs were produced and tested as previously published (13, 24).

At different timepoints, the animals were euthanized after blood collection and the spleens were harvested. Splenocytes were obtained and processed as described elsewhere (18). Serum was obtained from blood collected at the time points indicated in the Figures. Serum was separated by blood centrifugation for 5 minutes at 1,000 xg. The supernatant was collected and frozen at -20°C until use.

### Immunophenotyping

Splenic cDC1 and cDC2, as well as their expression of costimulatory molecules, were analyzed by flow cytometry. Five million splenocytes were first incubated with Fc Block (clone 2.4G2, BD Biosciences) for 15 minutes on ice and the cells were washed with FACs buffer (PBS 1x with 2% fetal bovine serum). Splenocytes were first incubated with the biotin-conjugated antibodies anti-CD19 (clone 1D3, BD Biosciences) and anti-CD3 (clone 145.2C11, BD Biosciences) for 40 minutes on ice in the dark. After washing, cells were incubated with anti-MHCII-Alexa fluor 700 (clone M5/114.15.2, I-A/I-E, ThermoFischer Scientific), anti-CD11c-BV421 (clone N418, BD Biosciences), anti-CD8α-BV786 (clone 53-67, BD Biosciences), anti-CD11b-PE.Cy7 (clone M1/70, ThermoFischer Scientific), anti-CD80-FITC (clone 16-10A1, BD Biosciences), anti-CD86-APC (clone GL1, BD Biosciences), anti-CD40-PE (clone 1C10, BD Biosciences) and streptavidin-APC.Cy7 (BD Biosciences), as well as with Aqua Live/Dead (ThermoFischer Scientific), for 40 minutes on ice in the dark.

To identify Tfh cells, 5x10^6^ splenocytes were incubated with anti-CXCR5-Biotin (clone 2G8, BD Biosciences) diluted in FACs buffer for 40-50 min at 37°C in the dark. After two washes, cells were incubated with anti-CD19-PE.Cy7 (clone 1D3, BD Biosciences), anti-CD3-APC.Cy7 (clone 145.2C11, BD Biosciences), anti-CD4-PerCP (clone RM4-5, BD Biosciences), anti-PD-1-APC (CD279, clone J43, BD Biosciences) and streptavidin-PE (BD Biosciences) including Aqua Live/Dead (Thermo Fischer Scientific) for 40 minutes on ice in the dark.

Germinal center and plasma cells were analyzed by flow cytometry. Five million (5x10^6^) splenocytes were incubated with anti-CD3-APC.Cy7 (clone 145.2C11, BD Biosciences), anti-B220-PerCP (clone RA3-6B2, BD Biosciences), anti-GL-7-FITC (clone GL7, BD Biosciences), anti-CD95-PE (clone Jo2, BD Biosciences), anti-CD138 (clone 281-2, BD Biosciences), and Aqua Live/Dead (Thermo Fischer Scientific) for 40 min on ice and in the dark.

### Intracellular analyses of pSTAT1 and pSTAT3

Five million splenocytes from WT, STAT3^Flox/Flox^ or STAT3 cKO mice were stimulated or not with a supernatant of WT splenocytes previously stimulated with anti-CD3 (clone 145.2C11, BD Biosciences) at 37°C for 15 or 20 minutes. After two washes with FACs buffer, cDC were labeled as described in the previous section, fixed and permeabilized using Phosflow III fixation buffer (BD Biosciences) for 10 minutes in a 37°C water bath. After washing twice with FACs buffer, cells were incubated with Phosflow I permeabilization buffer (BD Biosciences) for 30 minutes on ice and in the dark. Then, cells were washed twice and centrifuged at 500 xg for 5 minutes. pSTAT1 and/or pSTAT3 were labeled using the fluorochrome-conjugated antibodies anti-pSTAT1-PE-CF594 (clone 4a, BD Biosciences) and anti-pSTAT3-PE (clone J71-773.58.11, BD Biosciences) for 1 hour on ice in the dark.

### Intracellular cytokine staining

Intracellular cytokine staining was performed exactly as described elsewhere (13). Briefly, 1.5x10^6^ splenocytes were pulsed or not with 5 μg/mL of the recombinant MSP1_19_PADRE protein and 2 μg/mL of the anti-CD28 mAb (clone 37.51, BD Biosciences) in U-shaped 96-well plates (Costar). Cells stimulated only with 2 μg/mL of the anti-CD28 were used as negative controls. After incubation for one hour at 37°C and 5% CO_2_, 0.25 μg/well of Golgi Plug (Brefeldin A, BD Biosciences) was added to each well. Plates were incubated again at 37°C and 5% CO_2_ for 12 hours.

The intracellular cytokine staining was performed after an extracellular and viability staining with anti-CD4-PerCP (clone RM4-5, BD Biosciences) and Live and Dead Aqua (Thermo Fischer Scientific) for 30 minutes on ice. After two washes with FACS buffer, cells were fixed and permeabilized with Cytofix/Cytoperm kit (BD Biosciences), and washed twice using PermWash buffer (BD Biosciences). Finally, intracellular cytokine IFN-γ, IL-2, TNF-α, IL-10, and CD3 were stained with anti-IFNγ-APC (clone XMG1.2, BD Biosciences), anti-IL2-PE (clone JES6-5H4, BD Biosciences), anti-TNFα-PE-Cy7 (clone MP6-XT22, BD Biosciences), anti-IL-10-BV421 (clone JES5-16E3, BD Biosciences) and anti-CD3-APC-Cy7 (clone 145-2C11, BD Biosciences) on ice for 40 minutes. Then, cells were washed twice in FACS buffer. The frequencies of cytokine-producing cells were obtained subtracting the percent of cytokine-producing cells of the negative control wells from the stimulated samples.

For IL-21 staining in Tfh cells, 5x10^6^ splenocytes were stained for surface markers and viability, as previously described (24). After washing the cells twice with FACS buffer, cells were fixed and permeabilized using Cytofix/Cytoperm (BD Biosciences) for 15 minutes on ice, followed by washing with PermWash buffer (BD Biosciences). IL-21 was labeled using the anti-IL-21-PE (clone Mhalx21, eBioscience) for 1 hour on ice and in the dark. After two washes with FACS buffer, one million total cells were collected and analyzed.

### Intranuclear staining

Intranuclear expressions of Bcl-6, T-bet, and Ki67 were evaluated by flow cytometry. First, 5x10^6^ splenocytes were stained for surface markers and viability, as previously described. Then, cells were fixed and permeabilized with the FOXP3 labeling kit (eBioscience), according to the manufacturer’s instructions. Anti-Bcl6-PE (clone K112-91, BD Biosciences), and/or anti-T-bet-BV421 (clone O4-46, BD Biosciences), and/or KI67-BV421 (clone B56, BD Biosciences) were added and incubated for 1 hour on ice and in the dark. Cells were washed twice in FACS buffer and one million total events were acquired.

### Antibody titer

Specific anti-MSP1_19_ antibodies were detected by ELISA, as described elsewhere (13, 18). High binding 96 well plates (Costar) were coated with 2 μg/mL of recombinant MSP1_19_. Sera were serially diluted with a dilution factor of 3 starting at 1:100. Anti-mouse IgG conjugated with HRP (SouthernBiotech) were used to detect IgG anti-MSP1_19_ antibodies. IgG anti-MSP1_19_ antibody titers were considered as the highest serum dilution with an OD_490_ > 0.1 and normalized in a log10 scale.

### Migration of cDC1 *in vivo*

For the assessment of cDC1 migration *in vivo*, we used the labeling technique described by Calabro et al. that detects migration of cDC1 from the red to the white pulp (11). STAT3^Flox/Flox^ and STAT3 cKO mice received or not 50 µg of Poly (I:C) *via* the intraperitoneal route. After 6 hours, 1.5 µg of anti-CD11c-PE antibody (clone N418, BD Biosciences) was injected intravenously and mice were euthanized 3 minutes later. The spleen was removed, processed and 5x10^6^ splenocytes were labeled with fluorochrome-conjugated antibodies for the identification of cDC as described above. CD11c-PE^+^ cells were considered cDC1 that are located in the red pulp of the spleen.

### Data acquisition, absolute cell numbers, and statistical analysis

Flow cytometry data were acquired on a BD LSRFortessa™ Flow Cytometer (BD Biosciences) and analyzed using the FlowJo software (version 9.3, Tree Star, San Carlo, CA, USA). The gating strategy for Tfh cells, germinal center B cells, plasma cells, cytokine-producing cells, and for the expression of T-bet and Bcl-6 were performed exactly as described elsewhere (24). The absolute cell numbers were calculated according to the frequencies obtained in the analysis of the gating strategy of the flow cytometry results. Statistical analyses were performed in Prism 9.0 (GraphPad, CA, USA). One-way ANOVA followed by Tukey test was used for multiple comparisons, and Student's t-test was used for comparisons between two groups.

## Results

### Poly (I:C) triggers STAT3 phosphorylation in splenic cDC1 and cDC2

**Figure 1:**
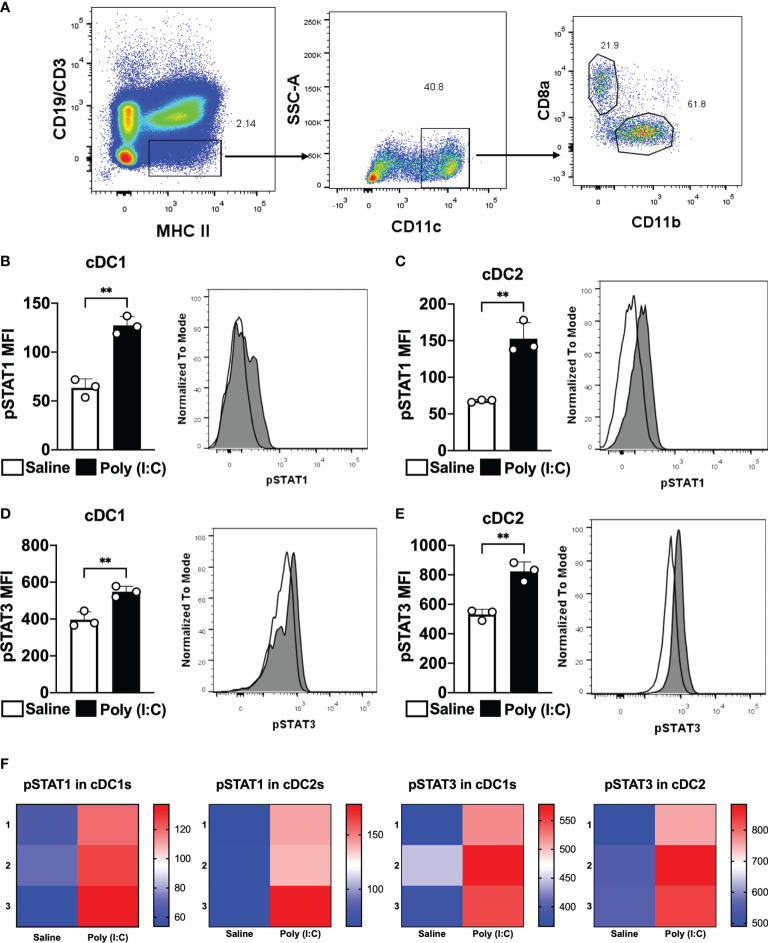
Poly (I:C) promotes STAT1 and STAT3 phosphorylation in cDC1 and cDC2. WT C57BL/6 mice were injected intraperitoneally with 50 μg of Poly (I:C) or only saline. Two hours later, splenocytes were obtained. Phosphorylated STAT1 (pSTAT1) and STAT3 (pSTAT3) were analyzed by phosflow. **(A)** Gating strategy to analyze splenic cDC1 and cDC2. Singlets and live cells were gated followed by CD19^-^CD3^-^MHCII^+^. Then, CD11c^+^ cells were selected. cDC1 were gated as CD8α^+^CD11b^-^ cells and cDC2 as CD8α^-^CD11b^+^. MFI for pSTAT1 is shown in cDC1 **(B)** and cDC2 **(C)**. MFI for pSTAT3 is shown in cDC1 **(D)** and cDC2 **(E)**. Histograms represent cells from mice injected with saline in white or Poly (I:C) in gray. **(F)** Heatmaps of pSTAT1 and pSTAT3 MFIs in cDC1 and cDC2 of 3 individual mice. Bars show mean ± SD from one representative experiment (n=3 animals/group) **p<0.01; Student's t-test.

In an attempt to study whether STAT3 signaling pathway controls cDC1 and cDC2 ability to prime CD4^+^ T cells, we started studying whether Poly (I:C) would promote STAT3 phosphorylation in cDC. As Poly (I:C) stimulates cDC *in vivo* mainly through interferon type I signaling, we also detected the phosphorylation of STAT1 (pSTAT1). pSTAT1 and pSTAT3 were detected in cDC1 and cDC2 by flow cytometry according to the strategy indicated in **Figure 1A**. After two hours of Poly (I:C) administration to mice, the MFI of pSTAT1 and pSTAT3 were analyzed. As expected, pSTAT1 MFI increased significantly in splenic cDC1 and cDC2 from mice that received Poly (I:C) when compared to the control group (saline only) (**Figures 1B, C, F**). Similarly, pSTAT3 MFI was higher in cDC1 and cDC2 from Poly (I:C)-injected mice than pSTAT3 MFI from non-stimulated mice (**Figures 1D–F**). These results indicate that Poly (I:C) triggers STAT1 and STAT3 phosphorylation in cDC1 and cDC2 *in vivo*.

### STAT3 does not control the development of cDC1 and cDC2

To study the influence of the STAT3 signaling pathway on cDC, we used CD11c^Cre^STAT3^Flox/Flox^ (STAT3 cKO) mice. In this system, STAT3 is deleted in CD11c^+^ cells which include cDC and their bone marrow progenitor cells. We analyzed whether the deletion of STAT3 gene in CD11c^+^ cells would impact the differentiation of splenic cDC1 or cDC2. We quantified total CD11c^+^ cells, cDC1 and cDC2 in the spleen of STAT3 cKO and STAT3^Flox/Flox^ mice by flow cytometry. The results showed that the STAT3 deletion in CD11c^+^ cells did not change the number (or frequency) of cDC (**Supplementary Figures 1A, B**), nor cDC1(**Supplementary Figures 1C, D**) and cDC2 (**Supplementary Figures 1E, F**), in the spleen of STAT3 cKO mice, suggesting that the STAT3 deletion in CD11c^+^ cells does not influence the differentiation of cDC, exactly as published previously (35).

In addition, to confirm that STAT3 cKO mice really delete STAT3 in cDC1 and cDC2, we performed an experiment to analyze STAT3 phosphorylation in these cells. Splenocytes from STAT3 cKO mice or STAT3^Flox/Flox^ were stimulated or not with the supernatant of WT splenocytes stimulated with anti-CD3 mAb, and pSTAT3 MFI was determined in cDC1, cDC2 and in CD4^+^ T cells as a control (**Supplementary Figures 2A, B**). The results showed that there was no statistically significant increase in pSTAT3 MFI in cDC1 and cDC2 from STAT3 cKO animals when compared to non-stimulated cells. pSTAT3 MFI was only increased in stimulated cDC1 and cDC2 from STAT3^Flox/Flox^ mice. It is important to point out that the pSTAT3 MFI of CD4^+^ T cells from both mouse strains was significantly higher in stimulated cells (**Supplementary Figures 2A, B**). Therefore, cDC1 and cDC2 from STAT3 cKO mice do not phosphorylate STAT3, as expected. Importantly, the absence of STAT3 signaling in CD11c^+^ cells did not alter STAT1 phosphorylation in cDC1s, cDC2s and CD4^+^ T cells (**Supplementary Figures 2C, D**).

### cDC2 promote Tfh cell responses in a STAT3-independent way after antigen targeting to the DCIR2 receptor

**Figure 2:**
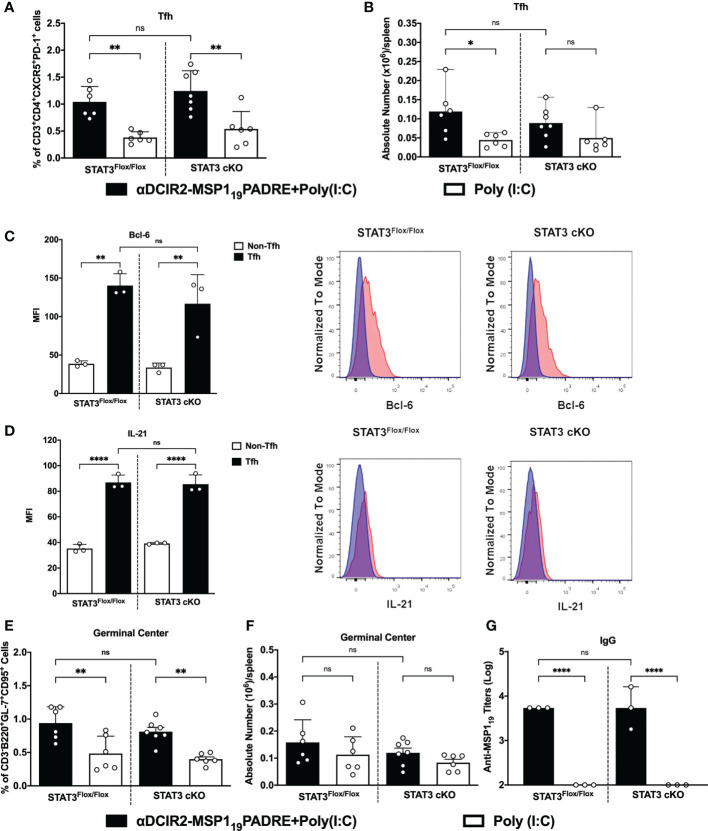
STAT3 signaling pathway does not control cDC2 ability to prime Tfh cells after antigen targeting to the DCIR2 receptor. STAT3^Flox/Flox^ and STAT3 cKO mice were immunized with 5μg of αDCIR2-MSP1_19_PADRE together with 50 μg of Poly (I:C) as adjuvant or only Poly (I:C) as a control. Splenocytes were obtained 5 days later and Tfh cells, GC B cells and plasma cells were analyzed by flow cytometry, and anti-MSP1_19_ IgG titers were determined by ELISA. **(A)** Frequency of Tfh cells in CD4^+^ T cells. **(B)** Absolute numbers of Tfh cells in the spleen. Bcl-6 and IL-21 were stained after fixation/permeabilization and their expression on Tfh cells was detected by flow cytometry. **(C)** MFI for Bcl-6 was determined in Tfh (CD3^+^CD4^+^CXCR5^High^PD-1^High^) and non-Tfh cells (CD3^+^CD4^+^CXCR5^-^PD-1^-^). **(D)** MFI for IL-21 was determined in Tfh and non-Tfh. Histograms in blue show non-Tfh and in red Tfh cells. **(E)** Frequency and **(F)** absolute numbers/spleen of germinal centers (GL7^+^CD95^+^CD3^-^B220^+^). **(G)** Sera were titrated to detect specific anti-MSP1_19_ IgG antibodies by ELISA. Bars show mean ± SD from two experiments pooled together (n=6-7 animals/group) in **(A, B, E, F)** and from one experiment (n=3 animals/group in **(C, D, G)**. *p<0.05, **p<0.01, ****p<0.0001, and ns not significant; one-way ANOVA followed by Tukey’s post-test.

To study the role of STAT3 signaling pathway in the control of cDC2-mediated immune responses, we took advantage of a strategy to deliver antigens to cDC2 *via* DCIR2 receptor. Previous results have shown that antigen targeting to cDC2 through the DCIR2 receptor induce Tfh cells and, consequently, humoral immune responses (21, 22, 24). In this way, we used the chimeric mAb αDCIR2 genetically linked to the MSP1_19_PADRE antigen and Poly (I:C) as adjuvant to target cDC2 *in vivo*. Mice were immunized and, five days later, the Tfh cell response promoted by antigen targeting to cDC2 in STAT3 cKO or in STAT3^Flox/Flox^ mice was analyzed (see gating strategy on **Supplementary Figure 3**). Our results confirmed that antigen targeting to cDC2 *via* the DCIR2 receptor promoted an increased frequency/absolute number of CXCR5^+^PD-1^+^CD4^+^ T cells in αDCIR2-MSP1_19_PADRE-immunized mice compared with the group that received only Poly (I:C). Interestingly, there was no significant difference in the frequency/absolute number of CXCR5^+^PD-1^+^CD4^+^ T cells between the STAT3 cKO and STAT3^Flox/Flox^ mice that were immunized with αDCIR2-MSP1_19_PADRE (**Figures 2A, B**).

To confirm that CXCR5^+^PD-1^+^ CD4^+^ T cells are indeed Tfh cells, we stained Bcl-6 intranuclearly and IL-21 intracellularly in those cells. Bcl-6 is the main transcription factor and IL-21 is the hallmark cytokine expressed in Tfh cells. Bcl-6 and IL-21 MFI were significantly higher in CXCR5^+^PD-1^+^ cells (Tfh cells) from STAT3 cKO and STAT3^Flox/Flox^ mice when compared to CXCR5^-^PD-1^-^ cells (non-Tfh cells). Again, in both cases, there was no significant difference when comparing the Bcl-6 and IL-21 MFI from Tfh cells from STAT3^Flox/Flox^ and STAT3 cKO mice (**Figures 2C, D**). Thus, these results indicate that STAT3 signaling pathway does not influence the ability of cDC2 to prime Tfh cells after antigen targeting *via* DCIR2 receptor.

Furthermore, germinal center (GC) formation, plasma cell differentiation (see gating strategy on **Supplementary Figure 4**) as well as antibody production were also evaluated after antigen targeting to cDC2 *via* DCIR2 receptor in STAT3 cKO or in STAT3^Flox/Flox^ mice. Both STAT3^Flox/Flox^ and STAT3 cKO mice that received αDCIR2-MSP1_19_PADRE had significantly higher frequencies and a tendence of higher GC B cell numbers (**Figures 2E, F**). Similarly, plasma cell frequency was significantly higher in all αDCIR2-MSP1_19_PADRE-immunized mice while the absolute numbers were higher in STAT3^Flox/Flox^ and there was a tendency of increase in STAT3 cKO mice (**Supplementary Figure 5**). Moreover, our results show that there were no significant differences in GC B cells (**Figures 2E, F**) and plasma cells (**Supplementary Figure 5**) between mice in which cDC signal or not *via* the STAT3 signaling pathway. When we determined anti-MSP1_19_ antibody titers in the serum, there was also no significant difference between the STAT3 cKO and STAT3^Flox/Flox^ mice (**Figure 2G**). Thus, the STAT3 signaling pathway in cDCs does not alter GC formation and plasma cell differentiation when antigen is targeted to cDC2 *via* DCIR2, nor antibody production. These results also confirm, once again, that antigen targeting to cDC2 *via* DCIR2 promotes GC formation and plasma cell differentiation. Taken together, these results suggest that cDC2s prime Tfh cells in a STAT3 independent manner after antigen targeting *via* DCIR2 receptor.

### STAT3 modulates the capacity of cDC1 to promote Th1 CD4^+^ T cell responses after antigen targeting to the DEC205 receptor

**Figure 3:**
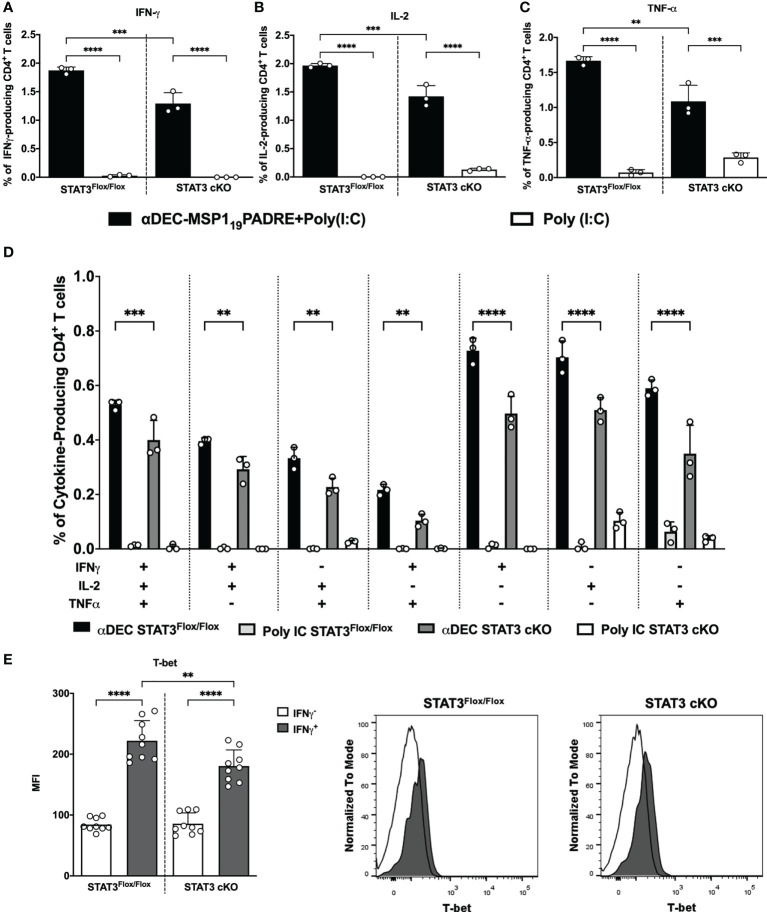
STAT3 signaling controls cDC1 ability to prime Th1 immune responses after antigen targeting to the DEC205 receptor. STAT3^Flox/Flox^ and STAT3 cKO mice were immunized with 5μg of αDEC205-MSP1_19_PADRE together with 50 μg of Poly (I:C) as adjuvant or only Poly (I:C) as a control. A boosting dose was performed 14 days later using only 5μg of αDEC205-MSP1_19_PADRE or only saline. Splenocytes were obtained 4 days after boosting. Splenocytes were stimulated ex vivo with 5μg/mL of recombinant MSP1_19_PADRE and incubated for 12 hours. Intracellular cytokine staining was performed after fixation and permeabilization. Graphs show the percentage of **(A)** IFN-γ-, **(B)** IL-2- and **(C)** TNF-α-producing CD4^+^ T cells. **(D)** Boolean gating on CD4^+^ T cells displaying polyfunctional cytokine-producing cells. **(E)** T-bet expression was determined in IFN-γ-producing CD4^+^ T cells. Histograms represent IFNγ^+^CD4^+^ T cells in black and IFNγ^-^CD4^+^ T cells in white. Bars show mean ± SD of one representative result out of 3 independent experiments (n=3 animals/group) in **(A–D)** and from three experiments pooled together (n=9 animals/group) in **(E)** **p<0.01, ***p<0.001, ****p<0.0001, and ns not significant; one-way ANOVA followed by Tukey’s post-test.

In an effort to elucidate whether the STAT3 signaling pathway plays a role in the modulation of cDC1 ability to prime Th1 CD4^+^ T cells responses after antigen targeting to the DEC205 receptor, STAT3^Flox/Flox^ and STAT3 cKO mice were immunized intraperitoneally with αDEC205-MSP1_19_PADRE using Poly (I:C) as adjuvant or with Poly (I:C) alone. Fourteen days after the first dose, the animals received a boosting dose using only αDEC205-MSP1_19_PADRE or saline. Four days after the second dose, the immune response promoted by antigen targeting to cDC1 *via* DEC205 receptor was accessed by intracellular cytokine staining to detect the production of the pro-inflammatory cytokines IFN-γ, IL-2 and TNF-α by CD4^+^ T cells (see gating strategy on **Supplementary Figure 6**). The flow cytometry results indicated that the frequencies of IFN-γ, IL-2 and TNF-α-producing CD4^+^ T cells were significantly higher in αDEC205-MSP1_19_PADRE-immunized mice when compared with the Poly (I:C) group. Besides, our results also showed that the frequencies of IFN-γ, IL-2 and TNF-α-producing CD4^+^ T cells were significatively reduced in STAT3 cKO mice compared to STAT3^Flox/Flox^ mice that received αDEC205-MSP1_19_PADRE (**Figures 3A–C**). In addition, there was a significant reduction in the frequency of cytokine-producing cells positive to three, two or one cytokine simultaneously (**Figure 3D**).

We also compared the promotion of Th1 response by cDC1s in groups of mice that received two doses of αDEC205-MSP1_19_PADRE in the presence or absence of Poly (I:C) (**Supplementary Figure 7**). As previously described (43, 44), antigen targeting to cDC1 through DEC205 in the absence of a maturation stimulus induces peripheral tolerance. In this way, the Th1 response induced in the absence of Poly (I:C) was severely reduced. Nonetheless, we still observed a significant reduction in the frequency of IFN-γ, IL-2 and TNF-α producing CD4^+^ T cells in STAT3 cKO mice when they were administered with αDEC205-MSP1_19_PADRE+Poly (I:C) (**Supplementary Figures 7A-C**). These results suggest that, upon activation with Poly (I:C), STAT3 signaling pathway stimulates cDC1 to promote pro-inflammatory CD4^+^ T cell response after antigen targeting *via* DEC205 receptor.

To confirm that IFN-γ-producing CD4^+^ T cells are Th1 cells, we stained T-bet intranuclearly on splenocytes from STAT3^Flox/Flox^ and STAT3 cKO mice, and analyzed the T-bet MFI on IFN-γ-producing CD4^+^ T cells (see gating strategy on **Supplementary Figure 6**). Results showed that the T-bet MFI on IFN-γ expressing CD4^+^ T cells (IFN-γ^+^) was significantly higher than the T-bet MFI on non-IFN-γ expressing cells, indicating that IFN-γ producing cells are Th1 cells. Additionally, T-bet MFI was significantly reduced in Th1 cells of STAT3 cKO compared to STAT3^Flox/Flox^ mice (**Figure 3E**). Taken together, our results indicate that, after antigen targeting to the DEC205 receptor in the presence of poly(I:C), the STAT3 signaling pathway stimulates cDC1 to prime Th1 CD4^+^ T cell responses *in vivo*.

### STAT3 signaling pathway stimulates DEC205 targeted cDC1 to prime T helper cell responses

The results described above were obtained when mice were immunized with two doses of the chimeric αDEC205-MSP1_19_PADRE mAb. In order to clarify if the reduction in the helper T cell responses observed in STAT3 cKO mice happened already during priming, we analyzed the CD4^+^ T cell pro-inflammatory response 14 days after the administration of a single dose of αDEC205-MSP1_19_PADRE in the presence of poly (I:C). The results showed that the frequencies of IFN-γ, IL-2 and TNF-α producing CD4^+^ T cells were significantly lower in STAT3 cKO mice when compared to STAT3^Flox/Flox^ mice (**Supplementary Figures 8A-C**). These results indicate that, once activated by poly (I:C), STAT3 signaling pathway stimulates cDC1 to prime Th1 CD4^+^ T cells after the first immunization with the αDEC205 mAb.

### STAT3 signaling pathway influences the frequency, but not the polarization, of Th1-like Tfh CD4^+^ T cells when the antigen is targeted to cDC1 through DEC205

**Figure 4:**
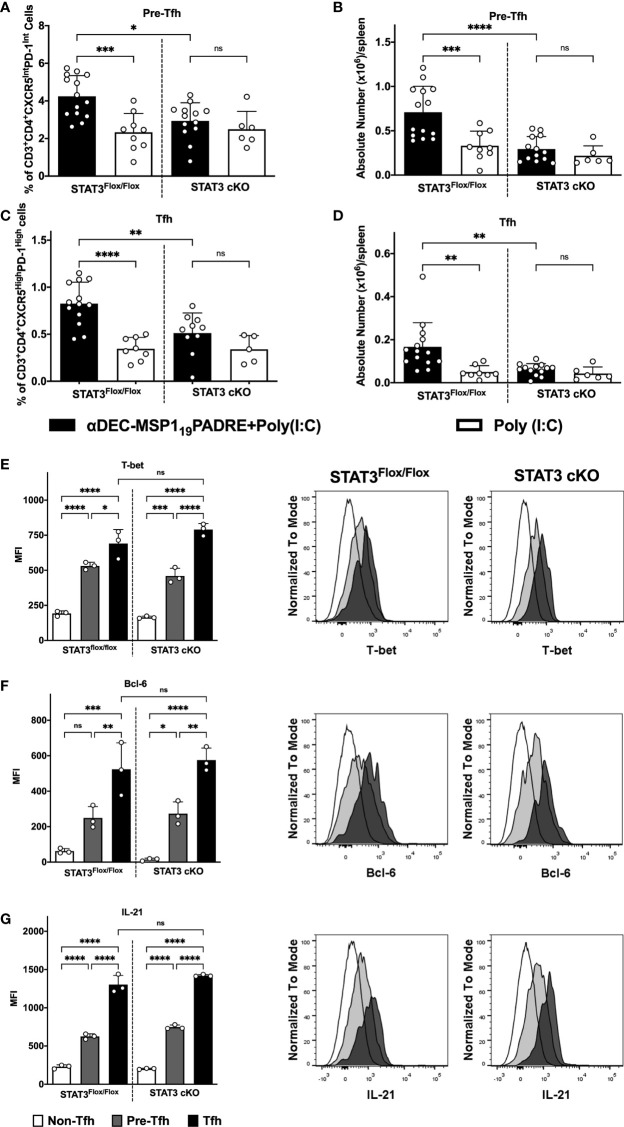
STAT3 signaling controls cDC1 ability to prime Tfh cells after antigen targeting to the DEC205 receptor. STAT3^Flox/Flox^ and STAT3 cKO mice were immunized as described in **Figure 3**. Splenocytes were obtained 4 days after boosting and Tfh cells were analyzed by flow cytometry. **(A)** Frequency and **(B)** absolute number of pre-Tfh cells (CD3^+^CD4^+^CXCR5^Inter^PD-1^Inter^) in the spleen. **(C)** Frequency **(D)** and absolute number of Tfh cells (CD3^+^CD4^+^CXCR5^High^PD-1^High^) in the spleen. Intracellular staining of Bcl-6 **(E)**, T-bet **(F)**, and IL-21 **(G)** in Tfh, pre-Tfh and non-Tfh cells (CD3^+^CD4^+^CXCR5^-^PD-1^-^) cells performed by flow cytometry in splenocytes 4 days after immunization. Histograms represent Non-Tfh cells in white, Pre-Tfh cells in gray and Tfh cells in black. Bars show mean ± SD from six experiments pooled together (n=5-14 animals/group) in **(A-D)** and one representative result out of 3 independent experiments (n=3 animals/group) in **(E–G)**. *p<0.05, **p<0.01, ***p<0.001, ****p<0.0001, and ns not significant; one-way ANOVA followed by Tukey’s post-test.

We have recently characterized a Th1-like Tfh CD4^+^ T cell response promoted after antigen targeting to cDC1 *via* DEC205 receptor, using the same immunization scheme described above. We showed that on day 4 after the administration of the second dose of αDEC205-MSP1_19_PADRE, we observed a very pronounced expansion of CXCR5^Int^PD-1^Int^CD4^+^ T cells (considered pre-Tfh cells) and CXCR5^High^PD-1^High^CD4^+^ T cells (Tfh cells) that expressed the Th1 transcription factor T-bet along with the Tfh hallmark Bcl-6 (24). In an attempt to study whether STAT3 deletion in cDC would also impact the Th1-like Tfh CD4^+^ T cell response promoted after antigen targeting *via* DEC205 receptor, we analyzed splenocytes of STAT3^Flox/Flox^ and STAT3 cKO mice 4 days after the administration of the second dose of αDEC205-MSP1_19_PADRE. We analyzed the frequency and the absolute numbers of pre-Tfh (CD3^+^CD4^+^CXCR5^Int^PD-1^Int^) and Tfh (CD3^+^CD4^+^CXCR5^High^PD-1^High^) cells (see gating strategy on **Supplementary Figure 9**). The results indicate that the frequency and the absolute numbers of pre-Tfh (**Figures 4A, B**) and Tfh (**Figures 4C, D**) CD4^+^ T cells were statistically increased in STAT3^Flox/Flox^ mice immunized with αDEC205-MSP1_19_PADRE. Interestingly, our results also indicated that both frequency and absolute number of pre-Tfh and Tfh cells were significantly reduced in STAT3 cKO mice when compared to STAT3^Flox/Flox^ mice that received αDEC205-MSP1_19_PADRE (**Figures 4A–D**). In summary, STAT3 signaling plays a role to stimulate DEC205 targeted cDC1 to promote Tfh cell responses.

To confirm that the CXCR5^High^PD-1^High^CD4^+^ T cells are indeed Tfh cells and correspond to Th1-like Tfh cells (see gating strategy on **Supplementary Figure 9**), we performed an intranuclear staining of transcription factors T-bet (**Figure 4E**) and Bcl-6 (**Figure 4F**), as well as the intracellular staining of IL-21 (**Figure 4G**). The MFI of T-bet, Bcl-6, and IL-21 were significantly higher in CXCR5^High^PD-1^High^. The medians of Bcl-6, T-bet and IL-21 fluorescence were also significantly higher in cells expressing intermediate levels of CXCR5 and PD-1 (CXCR5^Int^PD-1^Int^) when compared to the non-Tfh CD4^+^ T cell population. Furthermore, our results show that there were no significant differences when comparing the MFI of T-bet (**Figure 4E**), Bcl-6 (**Figure 4F**) and IL-21 (**Figure 4G**) between the STAT3^Flox/Flox^ and STAT3 cKO groups. Thus, these results indicate that the STAT3 signaling pathway does not influence polarization of Th1-like Tfh cell responses but seems to play a role in the induction of these cells.

### STAT3 depletion does not impact regulatory CD4^+^ T response nor Tfh cell proliferation after antigen targeting to cDC1 through DEC205

**Figure 5:**
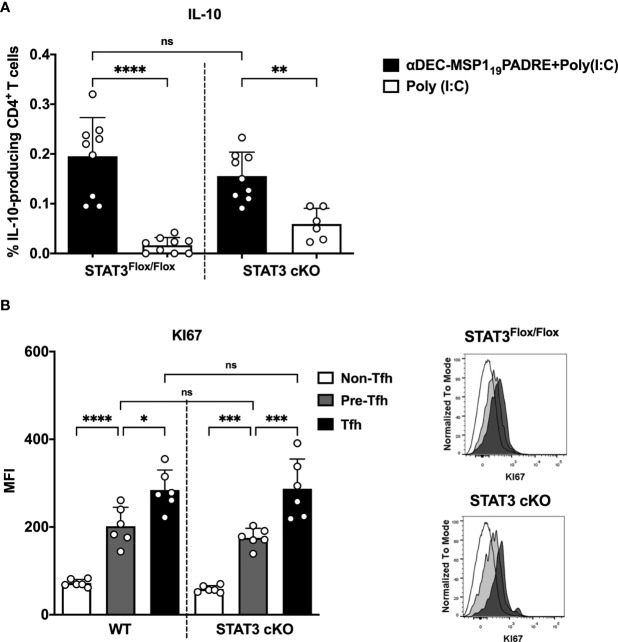
IL-10 production by CD4^+^ T cells is not influenced by STAT3 signaling pathway in cDC1 after antigen targeting to the DEC205 receptor. STAT3^Flox/Flox^ and STAT3 cKO mice were immunized as described in **Figure 3**. Splenocytes were obtained 4 days after boosting. **(A)** Intracellular cytokine staining for IL-10. Plots show the percentage of IL-10-producing CD4^+^ T cells. **(B)** Intracellular staining of Ki67 in Tfh, pre-Tfh and non-Tfh cells cells performed by flow cytometry in spleen cells 4 days after immunization. Histograms represent non-Tfh cells in white, pre-Tfh cells in gray and Tfh cells in black. Bars show mean ± SD from three experiments pooled together (n=9 animals/group, except STAT3cKO immunized with poly(I:C), n=6 animals/group) in **(A)**, and two experiments pooled together (n=6 animals/group) in **(B)** *p<0.05, **p<0.01, ***p<0.001, ****p<0.0001, and ns not significant; one-way ANOVA followed by Tukey’s post-test.

The experiments described above indicated that STAT3 depletion in DEC205 targeted cDC1 resulted in reduced Th1 and Th1-like Tfh cell responses. These results prompted us to evaluate if STAT3 depletion in cDC1 would impact the regulatory CD4^+^ T cell response that may repress the pro-inflammatory response. We performed intracellular cytokine staining to analyze the frequency of IL-10-producing CD4^+^ T cells. Surprisingly, there was no significant difference in the frequency of IL-10-producing CD4^+^ T cells when we compared STAT3^Flox/Flox^ and STAT3 cKO mice immunized with αDEC205-MSP1_19_PADRE (**Figure 5A**). Therefore, the decreased Th1 and Th1-like Tfh cell responses probably were not due to an increase in the frequency of IL-10 producing cells.

To rule out the possibility that CD4^+^ T cells from STAT3 cKO mice have any deficiency in their proliferation, we performed an intranuclear Ki67 staining in pre-Tfh and Tfh cells from STAT3^Flox/Flox^ and STAT3 cKO mice previously immunized with αDEC205-MSP1_19_PADRE+poly(I:C) (see gating strategy on **Supplementary Figure 9**). The results showed that the Ki67 MFI is statistically higher in Tfh and pre-Tfh cells when compared to non-Tfh CD4^+^ T cells, indicating that both subpopulations of Tfh cells were proliferating. When Ki67 MFI of Tfh cells was compared between STAT3^Flox/Flox^ and STAT3 cKO mice, there were no significant differences between these two groups (**Figure 5B**). Therefore, these results indicate that STAT3 cKO animals do not show any failure in the proliferation of Tfh cells, and the decreased number Tfh cells, and perhaps Th1 CD4^+^ cells (not tested), are not related to their capacity to proliferate *in vivo*.

### STAT3 does not control the expression of costimulatory molecules in cDCs targeted with DEC205 or DCIR2 in the presence of Poly (I:C)

In an attempt to understand the mechanism by which STAT3 stimulates DEC205 targeted cDC1 to promote Th1 and Tfh cell responses, we first analyzed whether the STAT3 deletion alters the maturation of cDCs *in vivo*. Thus, Poly (I:C) was administered intraperitoneally to STAT3^Flox/Flox^ and STAT3 cKO mice and, 24 hours later, we evaluated the expression of the costimulatory molecules CD80, CD86 and CD40 in splenic cDC1 and cDC2 (**Supplementary Figures 10A-F**). The results showed that the CD80, CD86 and CD40 MFI were significantly higher in all mice that received Poly (I:C) when compared to animals that received only saline (**Supplementary Figures 10A–E**), except when CD40 was compared in cDC2 (**Supplementary Figure 10F**). However, there was no significant difference between the MFI of any of the analyzed markers when comparing the STAT3^Flox/Flox^ and STAT3 cKO animals that received Poly (I:C) (**Supplementary Figures 10A-F**). Thus, these results show that the STAT3 signaling pathway *in vivo* does not influence the expression of costimulatory molecules CD80, CD86 and CD40 in splenic cDC1 and cDC2.

### STAT3 does not control cDC1 intrasplenic migration

In addition, we evaluated the intrasplenic migration of cDC1 by a method previously described (8). This technique consists of the intravenous administration of PE-conjugated anti-CD11c mAb for *in vivo* labeling of cDC1 located in the marginal region of the red pulp of the spleen. Three minutes after the administration of anti-CD11c-PE, the mice were euthanized. In this short period of time, anti-CD11c-PE can only penetrate the red pulp of the spleen, staining only the cDCs that are located in this region.

STAT3^Flox/Flox^ and STAT3 cKO mice received or not Poly (I:C) and, after 6 hours, the anti-CD11c-PE antibody was administered intravenously. CD11c-PE labeled cDC1 are located in the red pulp region and cDC2 in the marginal zone. The results indicated that mice that received Poly (I:C) showed a significant reduction in the frequency of CD11c-PE positive cDC1 when compared to mice that did not receive any agent that induces cDC maturation (naive mice) (**Supplementary Figures 11A, B**). On the contrary, cDC2 did not move (**Supplementary Figures 11A-C**). In this way, Poly (I:C) only induced the migration of cDC1 from the red pulp region, as the fewer cDC1 positive for CD11c-PE, the greater the number of cDC1 that migrated to the T cell zone. There was no significant difference when we compared the frequencies of CD11c-PE positive cDC1 and cDC2 between STAT3^Flox/Flox^ and STAT3 cKO mice that received Poly (I:C). This result indicates that the absence of STAT3 in cDC1 does not alter the migration of these cells. Thus, the reduced CD4^+^ T cell response in animals in STAT3 cKO mice occurs through a mechanism independent of the intrasplenic migration of this subtype of cDC1.

## Discussion

Dendritic cells are responsible to prime T cell responses, thus promoting immunity or tolerance in several circumstances (45). They are able to do so due to a division of labor between different subsets. cDC1 are specialized in cross-presentation of antigens and preferentially instruct CD4^+^ T cell responses to Th1 and Treg profiles (15, 23–25). On the other hand, cDC2 are primarily associated with their unique ability to induce Tfh and Th17 cell responses (22, 27, 46–48). Although each subtype is more specialized in promoting one or another type of CD4^+^ T cell response, it is known that cDC1 can also induce Tfh cell responses, just as cDC2 are capable of promoting Th1 and Treg responses (24, 26, 49). Nonetheless, mechanisms that regulate cDC1 and cDC2 function in order to produce distinct CD4^+^ T cell responses are still not fully understood.

Here, we decided to investigate the role of the STAT3 signaling pathway in the ability of cDC1 and cDC2 to prime CD4^+^ T cell responses after antigen targeting to these cells, in the presence of the adjuvant Poly (I:C), *via* the endocytic receptors DEC205 and DCIR2, respectively.

First, we analyzed whether Poly (I:C) administration would trigger the phosphorylation of STAT3 *in vivo*. Our data indicated that STAT3 was phosphorylated after two hours of Poly (I:C) injection in cDC1 and cDC2. Interestingly, STAT3 deletion did not alter STAT1 phosphorylation in cDCs. These results, combined with previous results showing that Poly (I:C) is an efficient adjuvant to increase the CD4^+^ T cell response after antigen targeting to cDC (15, 25), prompted us to use it to address the role of the STAT3 signaling pathway during cDC activation. In addition, Longhi et al. showed that the *in vivo* injection of Poly (I:C) promotes maturation of DC *via* type I interferon signaling (19). Accordingly, here we showed that Poly (I:C) induced STAT1 and STAT3 phosphorylation, and STAT1 and STAT3 are signaling pathways triggered by the type I interferon receptor (IFNAR1) (50). It is important to mention that Poly (I:C) can also induce type III interferons which have been reported to directly stimulate Th1 immune responses (51).

To study the influence of the STAT3 signaling pathway specifically in cDC, we used STAT3 cKO mice. Initially, we analyzed whether conditional knock out of STAT3 in CD11c^+^ cells would alter the differentiation of splenic cDC. Our results showed that STAT3 deficiency in CD11c^+^ cells did not impact the frequency or the absolute numbers of cDC1 and cDC2 in the mouse spleen, exactly as described previously (35). These results suggest that the STAT3 signaling pathway does not alter the differentiation of splenic cDC. It is known that STAT3 phosphorylation is essential for the differentiation of bone marrow precursor cells that give rise to cDC (52). However, these precursors do not yet express CD11c (53). As we used mice in which the STAT3 knock out happens conditioned to the expression of CD11c, the differentiation of cDC was not impaired in our model.

Furthermore, our results showed that STAT3 signaling pathway does not influence the ability of cDC2 to prime Tfh cells. For that, we targeted cDC2 *in vivo* using the αDCIR2-MSP1_19_PADRE together with Poly (I:C) as an adjuvant. After 5 days, we analyzed the immune response promoted by cDC2 in mice deficient or not for STAT3. The frequencies of Tfh cells, GC B cells, plasma cells and also the anti-MSP1_19_ antibody titers in STAT3 cKO mice were similar to the frequencies found in the control STAT3^Flox/Flox^ mice that are able to signal through the STAT3 pathway. Our results indicate that antigen targeting to cDC2 through DCIR2 and in the presence of Poly(I:C) promotes Tfh cell responses in a STAT3 independent manner. It is important to point out that B cells also play an important role in the response induced when mice are immunized with αDCIR2, as they also present antigens to T cells and promote their proliferation (47).

The influence of the STAT3 signaling pathway on the ability of cDC1 to promote T helper cell responses mediated by antigen targeting to DEC205 was also analyzed. For this, we performed two immunizations with αDEC205-MSP1_19_PADRE using Poly (I:C) as adjuvant, and analyzed the response 4 days after the second dose, exactly as described previously (24). We analyzed the responses of CD4^+^ T cells that are able to produce the cytokines IFN-γ, IL-2 and TNF-α (Th1) and Th1-like Tfh cells. The results showed a significant reduction of IFN-γ, IL-2 and TNF-α- producing CD4^+^ T cells (Th1), and Th1-like Tfh cells in STAT3 cKO mice. In fact, both Th1-like Tfh cell populations (mature Tfh and pre-Tfh) were significantly reduced in STAT3 cKO mice. Importantly, in our model, Poly (I:C) plays a fundamental role to promote Th1 immune responses as in its absence, the outcome of antigen targeting to DEC205^+^ cDC1 is tolerance (43, 44). These results indicated that STAT3 signaling pathway, once activated by Poly (I:C), stimulates cDC1 to promote Th1 and Th1-like Tfh cell responses after antigen targeting to cDC1 *via* DEC205 receptor.

As the data were analyzed after the administration of the second immunization, we decided to confirm that STAT3 signaling pathway influences the ability of DEC205^+^ cDC1 to initiate CD4^+^ T cell responses. So, we also addressed the frequency of IFN-γ, IL-2 and TNF-α- producing CD4^+^ T cells 14 days after the administration of the first dose of αDEC205-MSP1_19_PADRE together with Poly (I:C). The results showed that, again, the frequencies of IFN-γ, IL-2 and TNF-α producing cells were significantly lower in STAT3 cKO mice when compared to the STAT3^Flox/Flox^ group. Furthermore, our results of IL-10-producing CD4^+^ T cells and analysis of Tfh cell proliferation by Ki67 showed that the reduced Th1 and Th1-like Tfh cell response in STAT3 cKO mice did not occur due to an increased regulatory response, which could decrease the inflammatory response, nor due to a defect in the proliferation of T cells from STAT3 cKO mice. Thus, these results allow us to conclude that the STAT3 signaling pathway stimulates cDC1 to promote Th1 and Th1-like Tfh cell responses when the antigen is delivered to the DEC205 receptor and in the presence of Poly(I:C). Interestingly to point out that non-Tfh cells were not upregulating KI67. This result led us to hypothesize that Th1 cells may therefore be in the same gating of Tfh cells, thus suggesting that Tfh and Th1 cells are indeed Th1-like Tfh cells, as we previously reported (24).

In an effort to try to understand how STAT3 signaling pathway is involved in the induction of Th1 and Th1-like Tfh responses in our model, we analyzed the expression of the costimulatory molecules CD80, CD86 and CD40 in cDCs after Poly (I:C) injection *in vivo*. Our data showed that STAT3-deficient cDCs up-regulated the costimulatory molecules similarly to cDCs from STAT3^Flox/Flox^ mice. However, it is important to emphasize that the administration of Poly (I:C) *in vivo* promotes the maturation of cDC1 and cDC2, mainly through cytokine signaling. Although cDC1 express higher amounts of TLR3, which recognizes Poly (I:C), these cells are essentially matured *via* type I interferons after injection of Poly (I:C) *in vivo* (19, 25, 54). In contrast, previous data showed that cDC2, that do not express TLR3, are matured by TNF-α and IL-6 after Poly (I:C) injection that synergistically stimulate the upregulation of costimulatory molecules and, consequently, the migration of cDC2 from the intestinal mucosa in response to Poly (I:C) (54, 55). In our system, STAT3 deletion in splenic cDC2 did not inhibit CD80, CD86 and CD40 upregulation, nor altered their migration in the spleen, suggesting that another cytokine that does not trigger STAT3 activation may be playing this role. Furthermore, cDC2 preferentially signal *via* the non-canonical pathway of NF-Kb, and TNF-α signaling normally occurs through activation of the non-canonical of NF-Kb pathway in DCs (22, 56). Consequently, cDC2 are likely to mature *in vivo* by TNF-α-dependent mechanisms after mice receive Poly (I:C) *via* the intraperitoneal route. Further experiments would have to be performed to address this hypothesis.

In contrast to our results, *in vitro* blocking of STAT3 in monocyte-derived DCs decreased the upregulation of costimulatory markers and cytokine production after stimulation with LPS (57), indicating that STAT3 may play a role in the activation of DC *in vitro*. Our hypothesis is that STAT3 controls DEC205^+^ cDC1 ability to prime Th1 responses through type I interferon signaling. Type I interferons stimulate cDC1 to instruct Th1 responses and, consequently, interferon regulatory genes may be related to the maturation of cDC1 (19, 57). Ardouin et al. showed that, in the immunogenic maturation process induced by Poly (I:C) stimulation, cDC1 express IFN-stimulated genes (ISGs). Furthermore, when they compared genes that are differently expressed between cDC1 that were matured homeostatically or by the effect of Poly (I:C), they showed that the genes that are more expressed in Poly (I:C) stimulated cDC1 are related with type I interferon signaling and cytokine signaling pathways such as STAT1, STAT2, STAT3 and NF-Kb (39). However, further research is necessary to confirm the role of STAT3 on cDC1 maturation, as our study could not rule out the role of STAT3 signaling pathway in other CD11c-expressing cells to regulate Th1 and Tfh cell responses promoted after antigen targeting to cDC1 through DEC205.

Interestingly, our results showed that the STAT3 signaling pathway is important for the induction of helper T cell responses by cDC1 after antigen is targeted *via* the DEC205 receptor. However, cDC also tend to decrease their ability to induce tolerogenic responses in the absence of the STAT3 signaling pathway because they express higher levels of costimulatory molecules due to the loss of IL-10 signaling (35). Consequently, the data obtained in this study provide evidence that the STAT3 signaling pathway plays a dual role in controlling the functions of cDC, particularly cDC1, when the antigen is targeted to the DEC205 receptor. While STAT3 is responsible for maintaining tolerance, stimulating tolerogenic cDC1, it is also involved in the induction of inflammatory CD4^+^ T cell responses once cDC1 are matured in the presence of an inflammatory stimuli, such as Poly (I:C).

## Data availability statement

The original contributions presented in the study are included in the article/**Supplementary Material**. Further inquiries can be directed to the corresponding author.

## Ethics statement

The animal study was reviewed and approved by The Institutional Animal Care and Use Committee (IACUC) of the Institute of Biomedical Sciences of the University of São Paulo under the protocol number 7937100118, in agreement with the Brazilian national law on animal care (11.794/2008) and the ARRIVE guideline.

## Author contributions

FS: Conceptualization, Data curation, Formal analysis, Investigation, Validation, Methodology, Writing – original draft, Visualization. LM: Methodology, Validation. DS: Methodology. MY: Methodology. DR: Conceptualization, Methodology, Resources, Writing – review & editing. SB: Conceptualization, Methodology, Resources, Writing – review & editing, Visualization, Supervision, Project administration, Funding acquisition. All authors contributed to the article and approved the submitted version.

## Funding

This research was funded by the Sao Paulo Research Foundation (FAPESP, grant #2018/07142-9, 2017/17471-7 and 2014/50890-5), the Brazilian National Research Council (CNPq, grant #440721/2016-4), and the Coordination for the Improvement of Higher Level Personnel (CAPES, grant #2047/2016). FBS and LAM received fellowships from FAPESP (2018/00145–2 and 2018/20821–2, respectively). DSR and SBB received fellowships from CNPq.

## Acknowledgments

The authors would like to thank Danielle Cristina Gomes Chagas, Bruno de Castro Bertoldo and Anderson Domingos Silva for assistance in the animal facility.

## Conflict of interest

The authors declare that the research was conducted in the absence of any commercial or financial relationships that could be construed as a potential conflict of interest.

## Publisher’s note

All claims expressed in this article are solely those of the authors and do not necessarily represent those of their affiliated organizations, or those of the publisher, the editors and the reviewers. Any product that may be evaluated in this article, or claim that may be made by its manufacturer, is not guaranteed or endorsed by the publisher.

## References

[1] Steinman RM Hemmi H. Dendritic cells: translating innate to adaptive immunity. Curr Top Microbiol Immunol (2006) 311:17–58. doi: 10.1007/3-540-32636-7_2 17048704

[2] Ganguly D Haak S Sisirak V Reizis B. The role of dendritic cells in autoimmunity. Nat Rev Immunol (2013) 13:566–77. doi: 10.1038/nri3477 PMC4160805 23827956

[3] Amorim KNS Chagas DCG Sulczewski FB Boscardin SB. Dendritic cells and their multiple roles during malaria infection. J Immunol Res (2016) 2016: 2926436. doi: 10.1155/2016/2926436 27110574 PMC4823477

[4] Mellman I Steinman RM. Dendritic cells: specialized and regulated antigen processing machines. Cell (2001) 106:255–8. doi: 10.1016/s0092-8674(01)00449-4 11509172

[5] Patente TA Pinho MP Oliveira AA Evangelista GCM Bergami-Santos PC Barbuto JAM. Human dendritic cells: Their heterogeneity and clinical application potential in cancer immunotherapy. Front Immunol (2018) 9 3176:3176. doi: 10.3389/fimmu.2018.03176 30719026 PMC6348254

[6] Guilliams M Ginhoux F Jakubzick C Naik SH Onai N Schraml BU. Dendritic cells, monocytes and macrophages: a unified nomenclature based on ontogeny. Nat Rev Immunol (2014) 14:571–8. doi: 10.1038/nri3712 PMC4638219 25033907

[7] Eisenbarth SC. Dendritic cell subsets in T cell programming: location dictates function. Nat Rev Immunol (2019) 19:89–103. doi: 10.1038/s41577-018-0088-1 30464294 PMC7755085

[8] Calabro S Gallman A Gowthaman U Liu D Chen P Liu J. Bridging channel dendritic cells induce immunity to transfused red blood cells. J Exp Med (2016) 213:887–96. doi: 10.1084/jem.20151720 PMC4886363 27185856

[9] Dudziak D Kamphorst AO Heidkamp GF Buchholz VR Trumpfheller C Yamazaki S. Differential antigen processing by dendritic cell subsets *in vivo* . Science (2007) 315:107–11. doi: 10.1126/science.1136080 17204652

[10] Guilliams M Dutertre CA Scott CL McGovern N Sichien D Chakarov S. Unsupervised high-dimensional analysis aligns dendritic cells across tissues and species. Immunity (2016) 45:669–84. doi: 10.1016/j.immuni.2016.08.015 PMC5040826 27637149

[11] Calabro S Liu D Gallman A Nascimento MS Yu Z Zhang TT. Differential intrasplenic migration of dendritic cell subsets tailors adaptive immunity. Cell Rep (2016) 16:2472–85. doi: 10.1016/j.celrep.2016.07.076 PMC6323650 27545885

[12] Amorim KN Rampazo EV Antonialli R Yamamoto MM Rodrigues MM Soares IS. The presence of T cell epitopes is important for induction of antibody responses against antigens directed to DEC205(+) dendritic cells. Sci Rep (2016) 6:39250. doi: 10.1038/srep39250 28000705 PMC5175286

[13] Antonialli R Sulczewski FB Amorim K Almeida BDS Ferreira NS Yamamoto MM. CpG oligodeoxinucleotides and flagellin modulate the immune response to antigens targeted to CD8alpha(+) and CD8alpha(-) conventional dendritic cell subsets. Front Immunol (2017) 8:1727. doi: 10.3389/fimmu.2017.01727 29255470 PMC5723008

[14] Apostolico JS Lunardelli VAS Yamamoto MM Cunha-Neto E Boscardin SB Rosa DS. Poly(I:C) potentiates T cell immunity to a dendritic cell targeted HIV-multiepitope vaccine. Front Immunol (2019) 10:843. doi: 10.3389/fimmu.2019.00843 31105693 PMC6492566

[15] Bonifaz LC Bonnyay DP Charalambous A Darguste DI Fujii S Soares H. *In vivo* targeting of antigens to maturing dendritic cells *via* the DEC-205 receptor improves T cell vaccination. J Exp Med (2004) 199:815–24. doi: 10.1084/jem.20032220 PMC2212731 15024047

[16] Boscardin SB Hafalla JC Masilamani RF Kamphorst AO Zebroski HA Rai U. Antigen targeting to dendritic cells elicits long-lived T cell help for antibody responses. J Exp Med (2006) 203:599–606. doi: 10.1084/jem.20051639 16505139 PMC2118236

[17] Do Y Koh H Park CG Dudziak D Seo P Mehandru S. Targeting of LcrV virulence protein from yersinia pestis to dendritic cells protects mice against pneumonic plague. Eur J Immunol (2010) 40:2791–6. doi: 10.1002/eji.201040511 20812236

[18] Henriques HR Rampazo EV Goncalves AJ Vicentin EC Amorim JH Panatieri RH. Targeting the non-structural protein 1 from dengue virus to a dendritic cell population confers protective immunity to lethal virus challenge. PloS Negl Trop Dis (2013) 7:e2330. doi: 10.1371/journal.pntd.0002330 23875054 PMC3715404

[19] Longhi MP Trumpfheller C Idoyaga J Caskey M Matos I Kluger C. Dendritic cells require a systemic type I interferon response to mature and induce CD4+ Th1 immunity with poly IC as adjuvant. J Exp Med (2009) 206:1589–602. doi: 10.1084/jem.20090247 PMC2715098 19564349

[20] Rampazo EV Amorim KN Yamamoto MM Panatieri RH Rodrigues MM Boscardin SB. Antigen targeting to dendritic cells allows the identification of a CD4 T-cell epitope within an immunodominant trypanosoma cruzi antigen. PloS One (2015) 10:e0117778. doi: 10.1371/journal.pone.0117778 25679777 PMC4332658

[21] Shin C Han JA Choi B Cho YK Do Y Ryu S. Intrinsic features of the CD8alpha(-) dendritic cell subset in inducing functional T follicular helper cells. Immunol Lett (2016) 172:21–8. doi: 10.1016/j.imlet.2016.01.009 26850563

[22] Shin C Han JA Koh H Choi B Cho Y Jeong H. CD8alpha(-) dendritic cells induce antigen-specific T follicular helper cells generating efficient humoral immune responses. Cell Rep (2015) 11:1929–40. doi: 10.1016/j.celrep.2015.05.042 26095362

[23] Soares H Waechter H Glaichenhaus N Mougneau E Yagita H Mizenina O. A subset of dendritic cells induces CD4+ T cells to produce IFN-gamma by an IL-12-independent but CD70-dependent mechanism *in vivo* . J Exp Med (2007) 204:1095–106. doi: 10.1084/jem.20070176 PMC2118574 17438065

[24] Sulczewski FB Martino LA Almeida BDS Zaneti AB Ferreira NS Amorim K. Conventional type 1 dendritic cells induce TH 1, TH 1-like follicular helper T cells and regulatory T cells after antigen boost *via* DEC205 receptor. Eur J Immunol (2020) 50:1895–911. doi: 10.1002/eji.202048694 32673408

[25] Trumpfheller C Caskey M Nchinda G Longhi MP Mizenina O Huang Y. The microbial mimic poly IC induces durable and protective CD4+ T cell immunity together with a dendritic cell targeted vaccine. Proc Natl Acad Sci USA (2008) 105:2574–9. doi: 10.1073/pnas.0711976105 PMC2268178 18256187

[26] Yin X Chen S Eisenbarth SC. Dendritic cell regulation of T helper cells. Annu Rev Immunol (2021) 39:759–90. doi: 10.1146/annurev-immunol-101819-025146 33710920

[27] Krishnaswamy JK Gowthaman U Zhang B Mattsson J Szeponik L Liu D. Migratory CD11b(+) conventional dendritic cells induce T follicular helper cell-dependent antibody responses. Sci Immunol (2017) 2:eaam9169. doi: 10.1126/sciimmunol.aam9169 29196450 PMC7847246

[28] Liu D Yin X Olyha SJ Nascimento MSL Chen P White T. IL-10-Dependent crosstalk between murine marginal zone b cells, macrophages, and CD8alpha(+) dendritic cells promotes listeria monocytogenes infection. Immunity (2019) 51:64–76.e67. doi: 10.1016/j.immuni.2019.05.011 31231033 PMC6685086

[29] Silva MO Almeida BS Sales NS Diniz MO Aps L Rodrigues KB. Antigen delivery to DEC205(+) dendritic cells induces immunological memory and protective therapeutic effects against HPV-associated tumors at different anatomical sites. Int J Biol Sci (2021) 17:2944–56. doi: 10.7150/ijbs.57038 PMC8326119 34345218

[30] Murray PJ. The JAK-STAT signaling pathway: input and output integration. J Immunol (2007) 178:2623–9. doi: 10.4049/jimmunol.178.5.2623 17312100

[31] Rawlings JS Rosler KM Harrison DA. The JAK/STAT signaling pathway. J Cell Sci (2004) 117:1281–3. doi: 10.1242/jcs.00963 15020666

[32] Hillmer EJ Zhang H Li HS Watowich SS. STAT3 signaling in immunity. Cytokine Growth Factor Rev (2016) 31:1–15. doi: 10.1016/j.cytogfr.2016.05.001 27185365 PMC5050093

[33] Cheng F Wang HW Cuenca A Huang M Ghansah T Brayer J. A critical role for Stat3 signaling in immune tolerance. Immunity (2003) 19:425–36. doi: 10.1016/s1074-7613(03)00232-2 14499117

[34] Lunz JG 3rd Specht SM Murase N Isse K Demetris AJ. Gut-derived commensal bacterial products inhibit liver dendritic cell maturation by stimulating hepatic interleukin-6/signal transducer and activator of transcription 3 activity. Hepatology (2007) 46:1946–59. doi: 10.1002/hep.21906 17935227

[35] Melillo JA Song L Bhagat G Blazquez AB Plumlee CR Lee C. Dendritic cell (DC)-specific targeting reveals Stat3 as a negative regulator of DC function. J Immunol (2010) 184:2638–45. doi: 10.4049/jimmunol.0902960 PMC3099405 20124100

[36] Nefedova Y Cheng P Gilkes D Blaskovich M Beg AA Sebti SM. Activation of dendritic cells *via* inhibition of Jak2/STAT3 signaling. J Immunol (2005) 175:4338–46. doi: 10.4049/jimmunol.175.7.4338 PMC1351251 16177074

[37] Nefedova Y Huang M Kusmartsev S Bhattacharya R Cheng P Salup R. Hyperactivation of STAT3 is involved in abnormal differentiation of dendritic cells in cancer. J Immunol (2004) 172:464–74. doi: 10.4049/jimmunol.172.1.464 14688356

[38] Park SJ Nakagawa T Kitamura H Atsumi T Kamon H Sawa S. IL-6 regulates *in vivo* dendritic cell differentiation through STAT3 activation. J Immunol (2004) 173:3844–54. doi: 10.4049/jimmunol.173.6.3844 15356132

[39] Ardouin L Luche H Chelbi R Carpentier S Shawket A Montanana Sanchis F. Broad and largely concordant molecular changes characterize tolerogenic and immunogenic dendritic cell maturation in thymus and periphery. Immunity (2016) 45:305–18. doi: 10.1016/j.immuni.2016.07.019 27533013

[40] Rosa DS Tzelepis F Cunha MG Soares IS Rodrigues MM. The pan HLA DR-binding epitope improves adjuvant-assisted immunization with a recombinant protein containing a malaria vaccine candidate. Immunol Lett (2004) 92:259–68. doi: 10.1016/j.imlet.2004.01.006 15081621

[41] Caton ML Smith-Raska MR Reizis B. Notch-RBP-J signaling controls the homeostasis of CD8- dendritic cells in the spleen. J Exp Med (2007) 204:1653–64. doi: 10.1084/jem.20062648 PMC2118632 17591855

[42] Moh A Iwamoto Y Chai GX Zhang SS Kano A Yang DD. Role of STAT3 in liver regeneration: survival, DNA synthesis, inflammatory reaction and liver mass recovery. Lab Invest (2007) 87:1018–28. doi: 10.1038/labinvest.3700630 17660847

[43] Bonifaz L Bonnyay D Mahnke K Rivera M Nussenzweig MC Steinman RM. Efficient targeting of protein antigen to the dendritic cell receptor DEC-205 in the steady state leads to antigen presentation on major histocompatibility complex class I products and peripheral CD8+ T cell tolerance. J Exp Med (2002) 196:1627–38. doi: 10.1084/jem.20021598 PMC2196060 12486105

[44] Hawiger D Inaba K Dorsett Y Guo M Mahnke K Rivera M. Dendritic cells induce peripheral T cell unresponsiveness under steady state conditions *in vivo* . J Exp Med (2001) 194:769–79. doi: 10.1084/jem.194.6.769 PMC2195961 11560993

[45] Banchereau J Steinman RM. Dendritic cells and the control of immunity. Nature (1998) 392:245–52. doi: 10.1038/32588 9521319

[46] Briseno CG Satpathy AT Davidson J Ferris ST Durai V Bagadia P. Notch2-dependent DC2s mediate splenic germinal center responses. Proc Natl Acad Sci USA (2018) 115:10726–31. doi: 10.1073/pnas.1809925115 PMC6196531 30279176

[47] Chappell CP Draves KE Giltiay NV Clark EA. Extrafollicular b cell activation by marginal zone dendritic cells drives T cell-dependent antibody responses. J Exp Med (2012) 209:1825–40. doi: 10.1084/jem.20120774 PMC3457737 22966002

[48] Li J Lu E Yi T Cyster JG. EBI2 augments tfh cell fate by promoting interaction with IL-2-quenching dendritic cells. Nature (2016) 533:110–4. doi: 10.1038/nature17947 PMC4883664 27147029

[49] Kato Y Steiner TM Park HY Hitchcock RO Zaid A Hor JL. Display of native antigen on cDC1 that have spatial access to both T and b cells underlies efficient humoral vaccination. J Immunol (2020) 205:1842–56. doi: 10.4049/jimmunol.2000549 PMC7504891 32839238

[50] Tsai MH Pai LM Lee CK. Fine-tuning of type I interferon response by STAT3. . Front Immunol (2019) 10:1448. doi: 10.3389/fimmu.2019.01448 31293595 PMC6606715

[51] Zhou JH Wang YN Chang QY Ma P Hu Y Cao X. Type III interferons in viral infection and antiviral immunity. Cell Physiol Biochem (2018) 51:173–85. doi: 10.1159/000495172 30439714

[52] Laouar Y Welte T Fu XY Flavell RA. STAT3 is required for Flt3L-dependent dendritic cell differentiation. Immunity (2003) 19:903–12. doi: 10.1016/s1074-7613(03)00332-7 14670306

[53] Naik SH Sathe P Park HY Metcalf D Proietto AI Dakic A. Development of plasmacytoid and conventional dendritic cell subtypes from single precursor cells derived *in vitro* and *in vivo* . Nat Immunol (2007) 8:1217–26. doi: 10.1038/ni1522 17922015

[54] Edwards AD Diebold SS Slack EM Tomizawa H Hemmi H Kaisho T. Toll-like receptor expression in murine DC subsets: lack of TLR7 expression by CD8 alpha+ DC correlates with unresponsiveness to imidazoquinolines. Eur J Immunol (2003) 33:827–33. doi: 10.1002/eji.200323797 12672047

[55] Garcias Lopez A Bekiaris V Muller Luda K Hutter J Ulmert I Getachew Muleta K. Migration of murine intestinal dendritic cell subsets upon intrinsic and extrinsic TLR3 stimulation. Eur J Immunol (2020) 50:1525–36. doi: 10.1002/eji.201948497 32383212

[56] Jie Z Yang JY Gu M Wang H Xie X Li Y. NIK signaling axis regulates dendritic cell function in intestinal immunity and homeostasis. Nat Immunol (2018) 19:1224–35. doi: 10.1038/s41590-018-0206-z PMC6195481 30250187

[57] De Giovanni M Cutillo V Giladi A Sala E Maganuco CG Medaglia C. Spatiotemporal regulation of type I interferon expression determines the antiviral polarization of CD4(+) T cells. Nat Immunol (2020) 21:321–30. doi: 10.1038/s41590-020-0596-6 PMC7043938 32066949

