# Supplementary material for: STAT3 signaling modulates the immune response induced after antigen targeting to conventional type 1 dendritic cells through the DEC205 receptor
Source: Front Immunol. 2022 Oct 18;13:1006996. doi: 10.3389/fimmu.2022.1006996 (PMC9624190; doi:10.3389/fimmu.2022.1006996)
Supplement: Supplementary file 1 [file DataSheet_1.pdf]

Supplementary Material

STAT3 Signaling Modulates the Immune Response Induced After Antigen Targeting to Conventional Type 1 Dendritic Cells through the DEC205 receptor

Fernando Bandeira Sulczewski[1], Larissa Alves Martino[1], Davi Salles[1], Márcio Massao Yamamoto[1], Daniela Santoro Rosa[2,3], Silvia Beatriz Boscardin[1,3]*

[1] Departamento de Parasitologia, Instituto de Ciencias Biomedicas, Universidade de Sao Paulo, Sao Paulo, Brazil

[2] Departamento de Microbiologia, Imunologia e Parasitologia, Universidade Federal de Sao Paulo, Sao Paulo, Brazil

[3] Instituto de Investigação em Imunologia (iii), INCT, Sao Paulo, Brazil

*** Correspondence:**

Dr Silvia Beatriz Boscardin

sbboscardin@usp.br

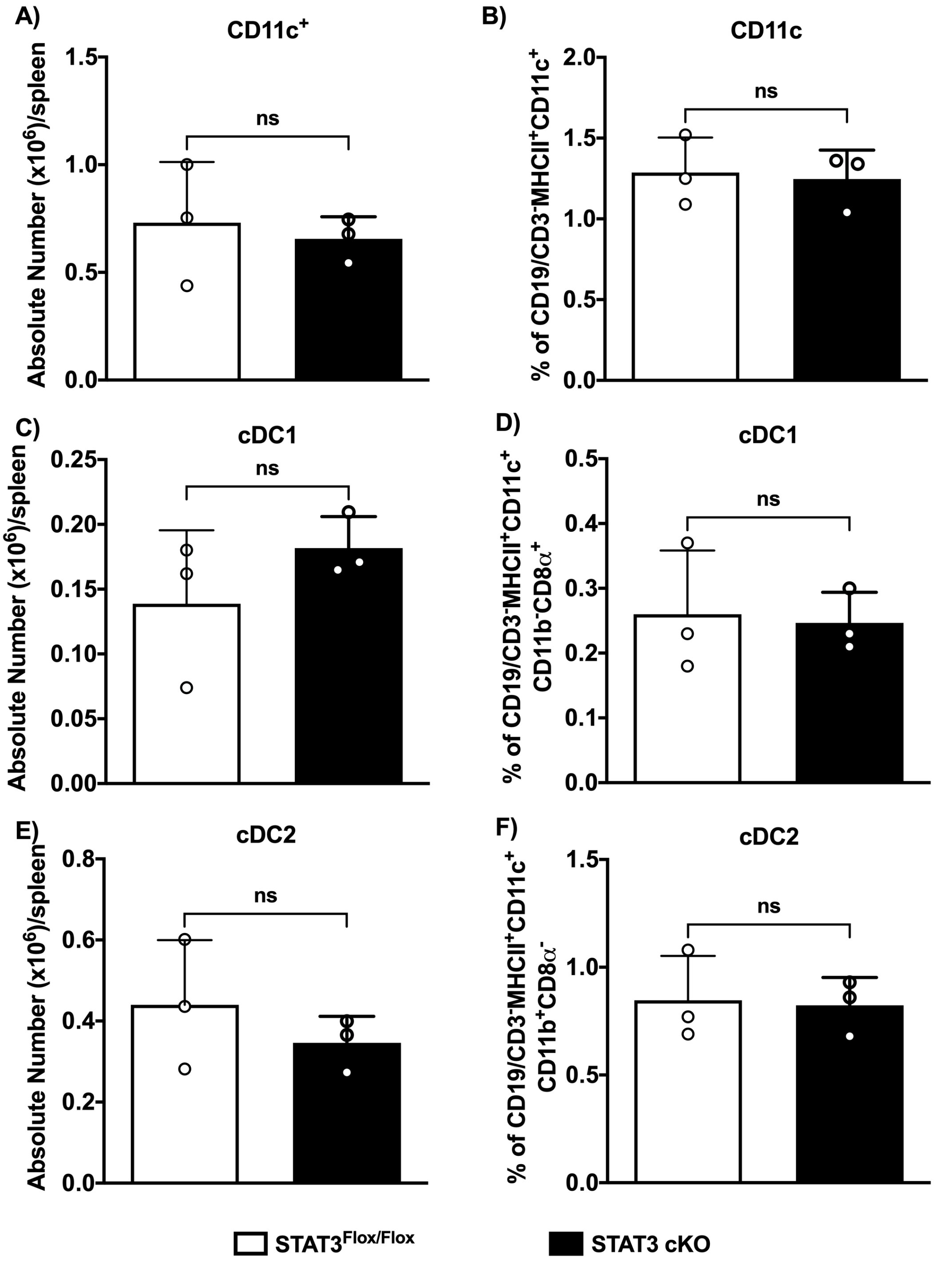

SUPPLEMENTARY FIGURE 1 The absence of STAT3 signaling does not alter the number of cDC in the spleen. Splenocytes from STAT3$^{Flox/Flox}$ and STAT3 cKO mice were obtained. Splenic DCs were quantified by flow cytometry. **(A)** Absolute numbers and **(B)** frequency of CD11c^{+} cells. **(C)** Absolute numbers and **(D)** frequency cDC1s. **(E)** Absolute numbers and **(F)** frequency of cDC2s. Unpaired t-test. Bars show mean±SD from one experiment (n=3 animals/group). ns, not significant.

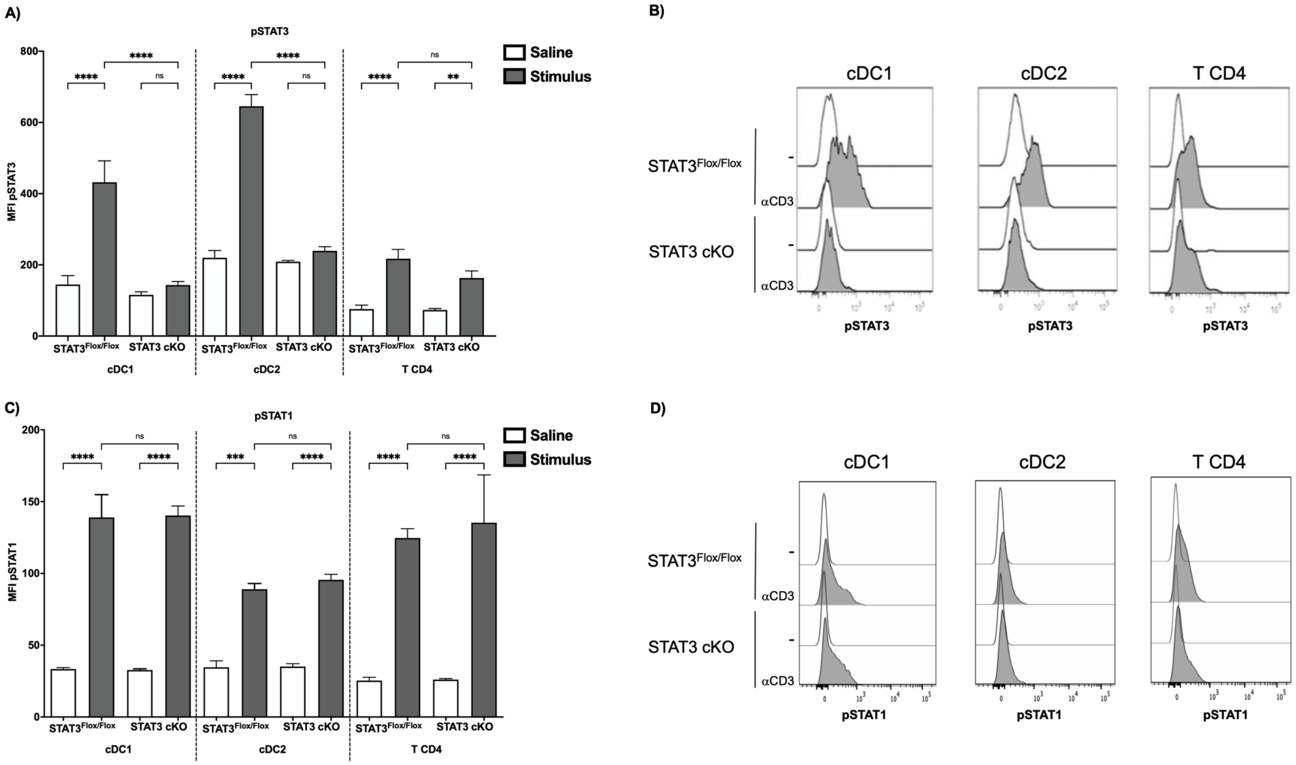

SUPPLEMENTARY FIGURE 2 Splenocytes from STAT3 cKO do not phosphorylate STAT3 in cDC1 and cDC2. Splenocytes were stimulated (grey) or not (white) with the supernatant of WT splenocytes previously treated with anti-CD3. Cells were labeled, fixed and permeabilized using BD Phosflow Perm buffers I and III according to the manufacturer's instructions. **(A)** MFI of pSTAT3 in cDC1s, cDC2s and $CD4^+$ T cells. **(B)** Histograms show unstimulated cells in white and stimulated cells in grey. **(C)** MFI of pSTAT1 in cDC1s, cDC2s and $CD4^+$ T cells. **(D)** Histograms show unstimulated cells in white and stimulated cells in grey. One-way analysis of variance (one-way ANOVA) was applied, followed by Tukey's test. Bars show mean±SD from one experiment (n=3 animals/group). **p<0.01, ****p<0.0001 and ns, not significant.

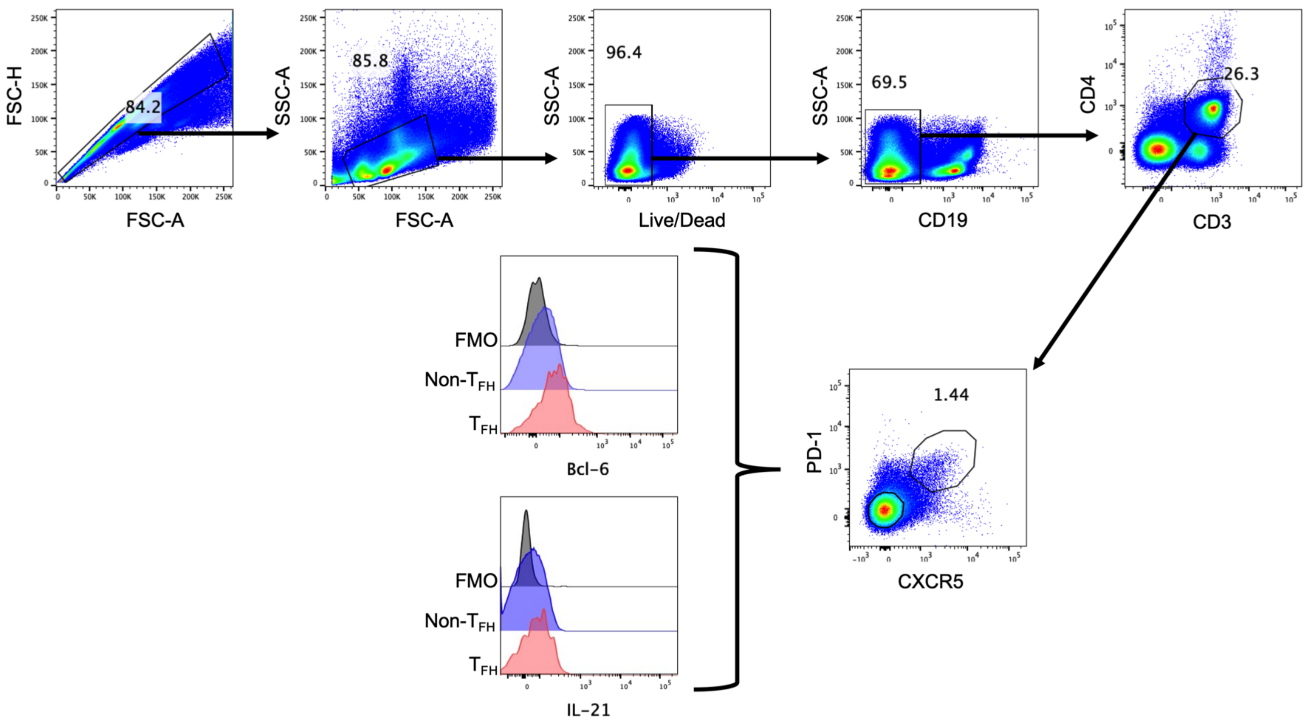

SUPPLEMENTARY FIGURE 3 Gating strategy to analyze Tfh cells induced by immunization with αDCIR2-MSP1$_{19}$PADRE. Mice were immunized with the chimeric αDCIR2-MSP1$_{19}$PADRE mAb as described in Figure 2. Splenocytes were obtained and stained with fluorochrome-conjugated antibodies. After sample acquisition in the flow cytometer, cells were gated in singlets (FSC-A x FSC-H) followed by size x granularity (FSC-A x SSC-A), live cells, CD19^{-}, and CD3^{+}CD4^{+}. For the Tfh staining, cells were gated on CXCR5^{+}PD-1^{+} and non-Tfh were gated on CXCR5^{-}PD-1^{-}. Histograms were used for Bcl-6 and IL-21 MFI calculation. FMO, fluorescence minus one.

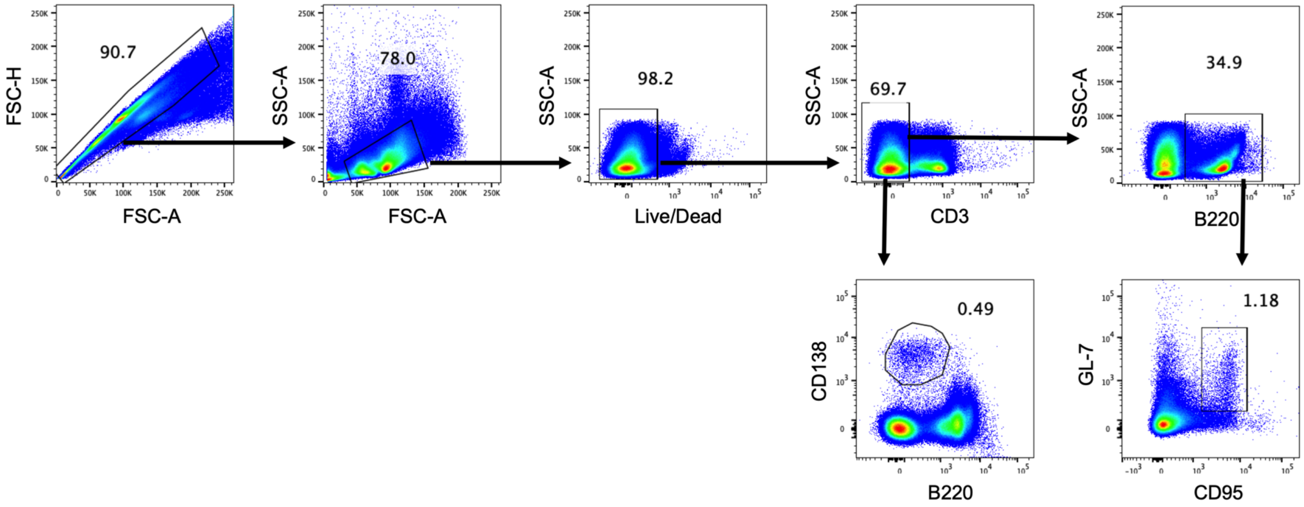

SUPPLEMENTARY FIGURE 4 Gating strategy to analyze germinal center B cells and plasma cells induced after immunization with αDCIR2-MSP1$_{19}$PADRE. Mice were immunized with the chimeric αDCIR2-MSP1$_{19}$PADRE mAb as described in Figure 2. Splenocytes were obtained and stained with fluorochrome-conjugated antibodies. After sample acquisition in the flow cytometer, cells were gated in singlets (FSC-A x FSC-H) followed by size x granularity (FSC-A x SSC-A), live cells, and CD3^{-}. Then, for germinal center B cells, B220^{+} cells followed by GL-7^{+}CD95^{+} cells or for plasma cells B220^{Low}CD138^{High}.

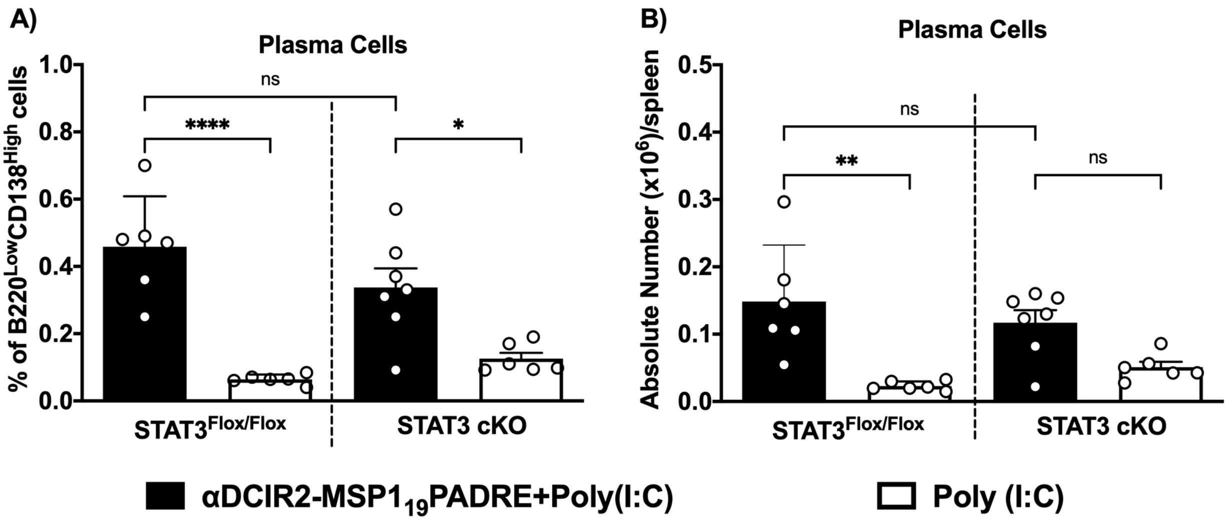

SUPPLEMENTARY FIGURE 5 STAT3 signaling pathway in cDCs does not control plasma cell differentiation after antigen targeting to cDC2s via DCIR2 receptor. $STAT3^{Flox/Flox}$ and STAT3 cKO mice were immunized as described in Figure 2. Splenocytes were obtained 5 days later and plasma cells were analyzed by flow cytometry. **(A)** Frequency and **(B)** absolute numbers of plasma cells ($CD3^{-}$ $B220^{Low}CD138^{High}$) in the spleen. Bars show mean±SD from two experiments pooled together (n=6-7 animals/group). *p<0.05, **p<0.01, ****p<0.0001, and ns, not significant; one-way ANOVA followed by Tukey's post-test.

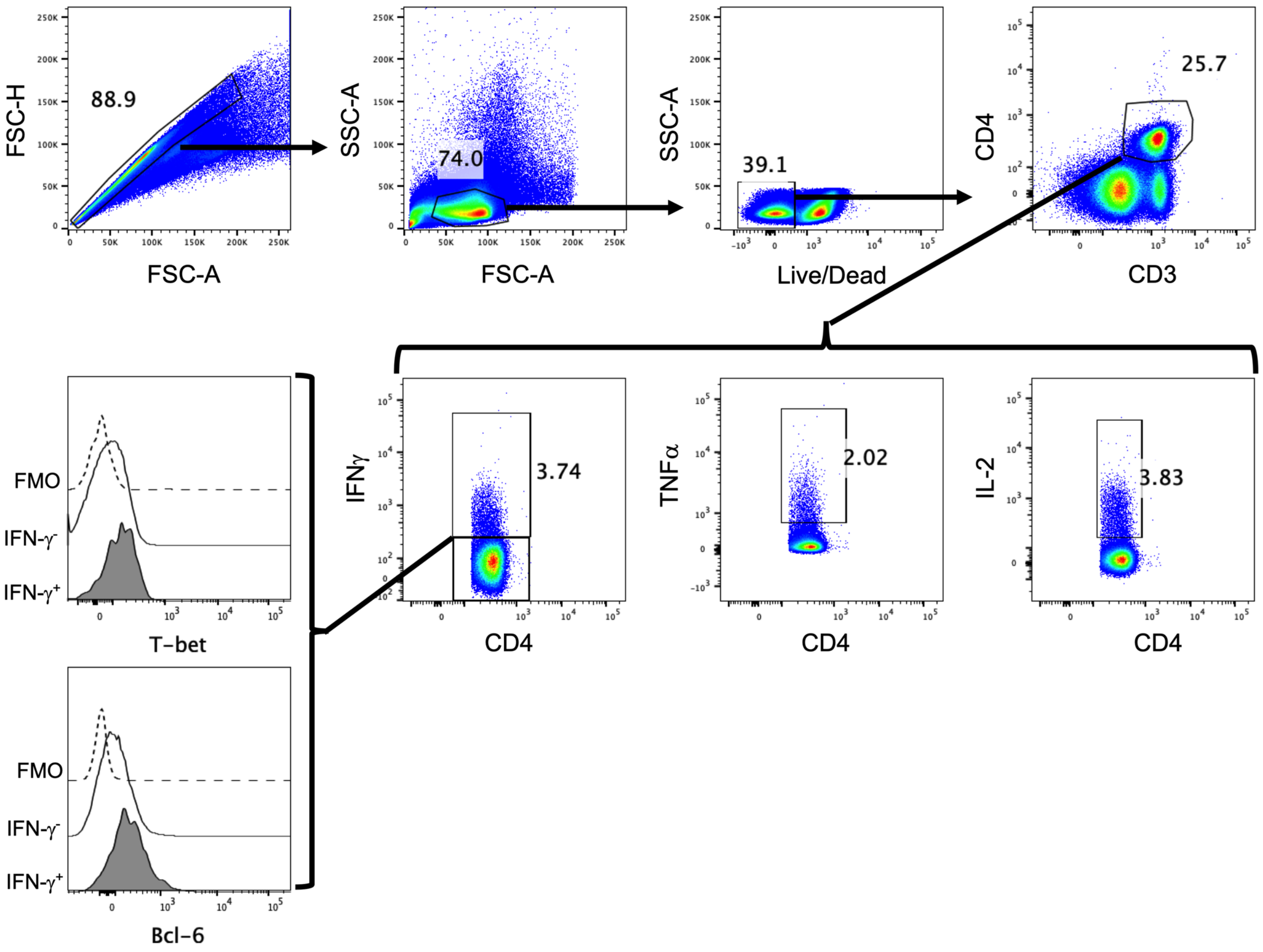

SUPPLEMENTARY FIGURE 6 Gating strategy to analyze CD4+ T cells producing IFNγ, TNFα and IL-2 induced after immunization with αDEC205-MSP1$_{19}$PADRE. Mice were immunized with the chimeric αDEC205-MSP1$_{19}$PADRE mAb as described in Figure 3. Splenocytes were obtained and stained with fluorochrome-conjugated antibodies. After sample acquisition in the flow cytometer, cells were gated in singlets (FSC-A x FSC-H) followed by size x granularity (FSC-A x SSC-A), live cells, $CD3^+CD4^+$, and finally $CD4^+IFN\gamma^+$, $CD4^+IL\text{-}2^+$, $CD4^+TNF\alpha^+$. The MFI of T-bet and Bcl-6 were further analyzed in the $CD4^+IFN\gamma^+$ and $CD4^+IFN\gamma^-$. FMO, fluorescence minus one.

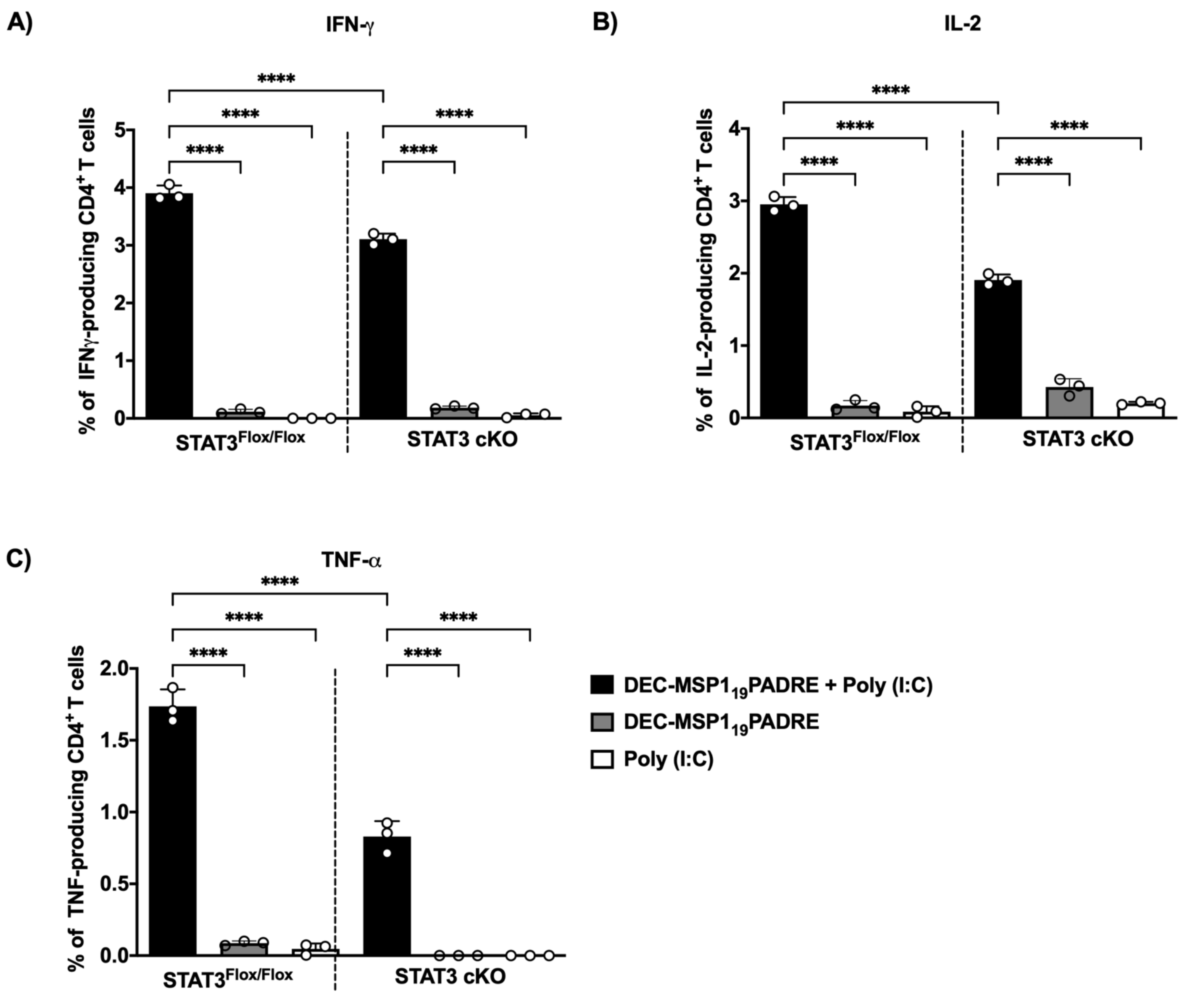

SUPPLEMENTARY FIGURE 7 cDC1s required a maturation stimulus to promote Th1 immune response after antigen targeting though DEC205 receptor. STAT3$^{Flox/Flox}$ and STAT3 cKO mice were immunized as described in figure 3 with the following groups: 5µg of αDEC205-MSP1$_{19}$PADRE together with 50 µg of Poly (I:C) as adjuvant, αDEC205-MSP1$_{19}$PADRE without poly(I:C) or only Poly (I:C), as a control. CD4^{+} T cell immune response was analyzed by intracellular cytokine staining 14 days after immunization. Graphs show the percentage of **(A)** IFN-γ-, **(B)** IL-2- and **(C)** TNF-α-producing CD4^{+} T cells. Bars show mean±SD from one experiment (n=3 animals/group). ****$p<0.0001$; one-way ANOVA followed by Tukey's post-test.

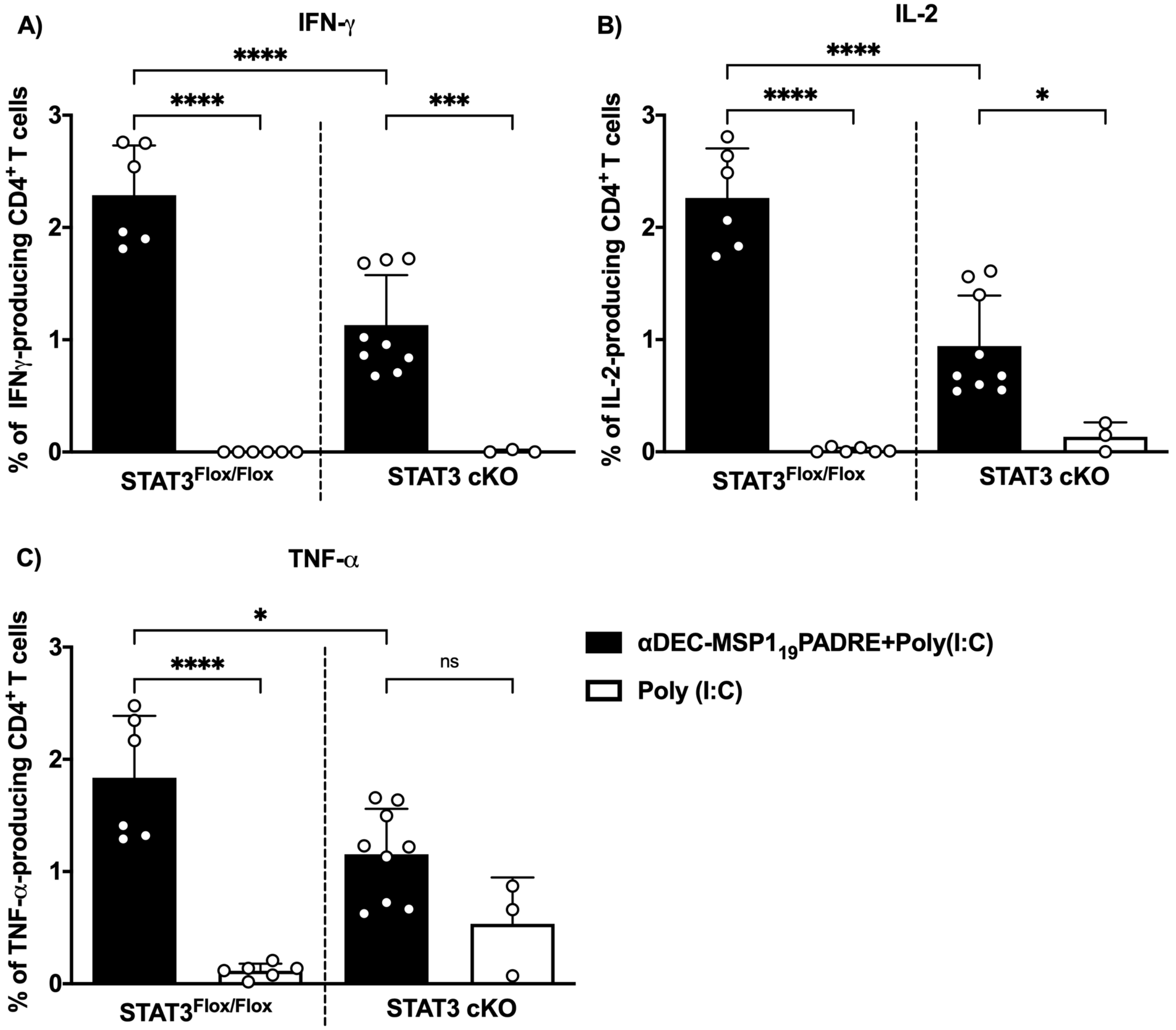

SUPPLEMENTARY FIGURE 8 STAT3 signaling pathway controls cDC1 ability to prime CD4+ T cell responses after antigen targeting to the DEC205 receptor. $STAT3^{Flox/Flox}$ and STAT3 cKO mice were immunized with 5μg of αDEC205-$MSP1_{19}$PADRE together with 50 μg of Poly (I:C) as adjuvant or only Poly (I:C) as a control. $CD4^+$ T cell immune response was analyzed by intracellular cytokine staining 14 days after immunization exactly as described in the figure 3. Graphs show the percentage of **(A)** IFN-γ-, **(B)** IL-2- and **(C)** TNF-α-producing $CD4^+$ T cells. Bars show mean±SD from two experiments pooled together (n=6 animals/group, except for STAT3 cKO immunized with Poly(I:C), n=3 animals/group). *p<0.05, ***p<0.001, ****p<0.0001, and ns, not significant; one-way ANOVA followed by Tukey's post-test.

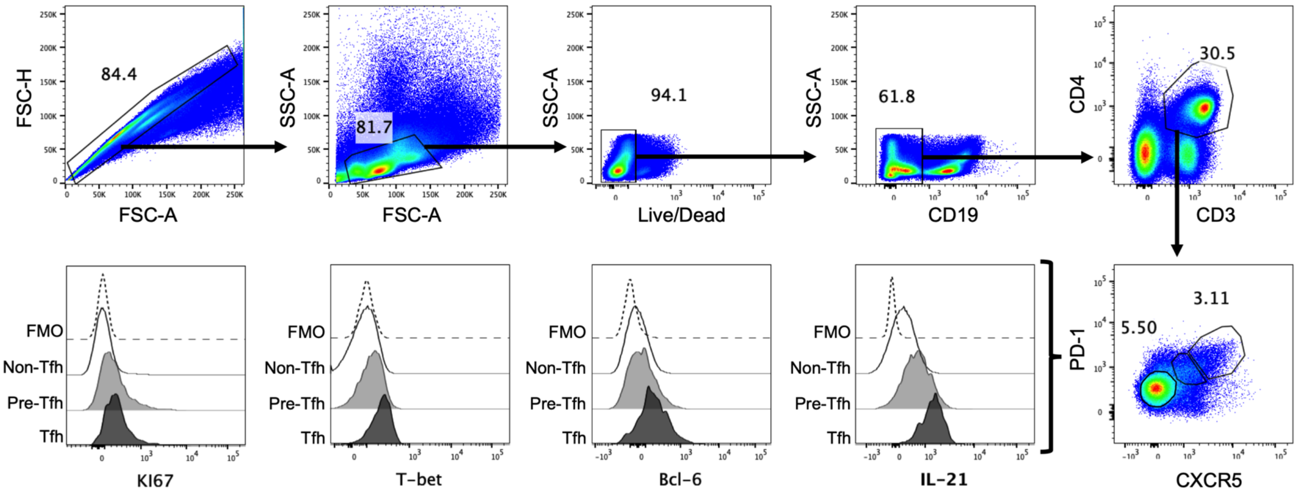

SUPPLEMENTARY FIGURE 9 Gating strategy to analyze T follicular helper cells (Tfh cells) induced after immunization with αDEC205-MSP1$_{19}$PADRE. Mice were immunized with the chimeric αDEC205-MSP1$_{19}$PADRE mAb as described in Figure 3. Four days after the administration of the boost, splenocytes were obtained and stained with fluorochrome-conjugated antibodies. After sample acquisition in the flow cytometer, cells were gated in singlets (FSC-A x FSC-H) followed by size x granularity (FSC-A x SSC-A), live cells, and CD3^{+}CD4^{+}. CXCR5^{High}PD-1^{High} and CXCR5^{Int}PD-1^{Int} cells were considered Tfh and pre-Tfh, respectively. CXCR5^{-}PD-1^{-} were considered non-Tfh cells. The MFI of KI67, T-bet, Bcl-6 and IL-21 were further analyzed in Tfh, pre-Tfh and non-Tfh cells. FMO, fluorescence minus one.

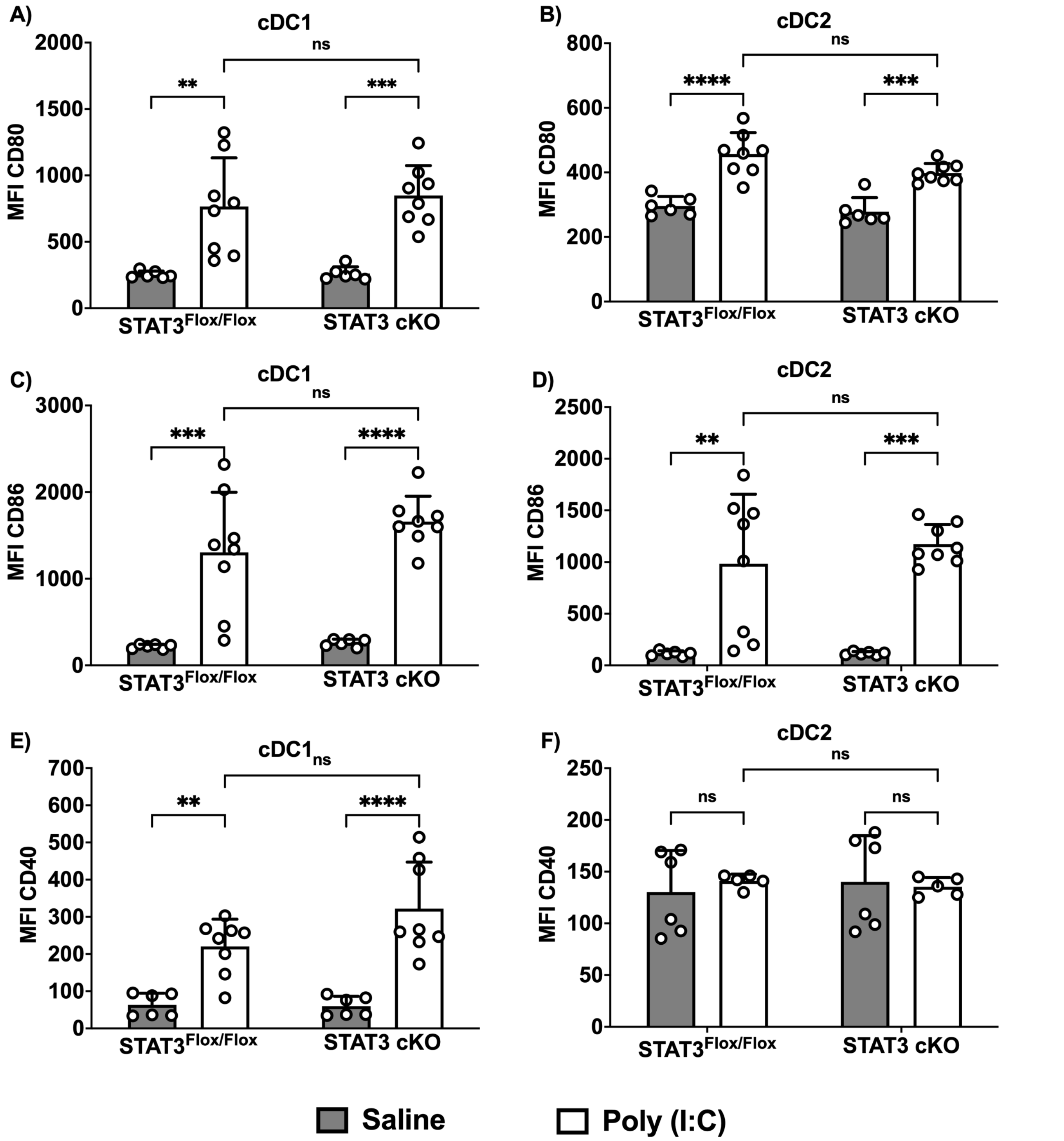

SUPPLEMENTARY FIGURE 10 STAT3 signaling does not impact costimulatory molecules. Fifty (50) µg of Poly (I:C) were injected intraperitoneally in STAT3$^{Flox/Flox}$ and STAT3 cKO mice. Six hours after Poly (I:C) administration, splenocytes were obtained and analyzed by flow cytometry. cDC1 were gated as CD11c^{+}MHCII^{+}CD8α^{+}CD11b^{-} cells and cDC2 as CD11c^{+}MHCII^{+}CD8α^{-}CD11b^{+} cells. The median of fluorescence intensity (MFI) for CD80, CD86 and CD40 was analyzed in cDC1 **(A-C-E)** and cDC2 **(B-D-F)**. Bars show mean±SD from two experiments pooled together (n=5-8 animals/group). **p<0.01, ***p<0.001, ****p<0.0001, and ns not significant; one-way ANOVA followed by Tukey's post-test.

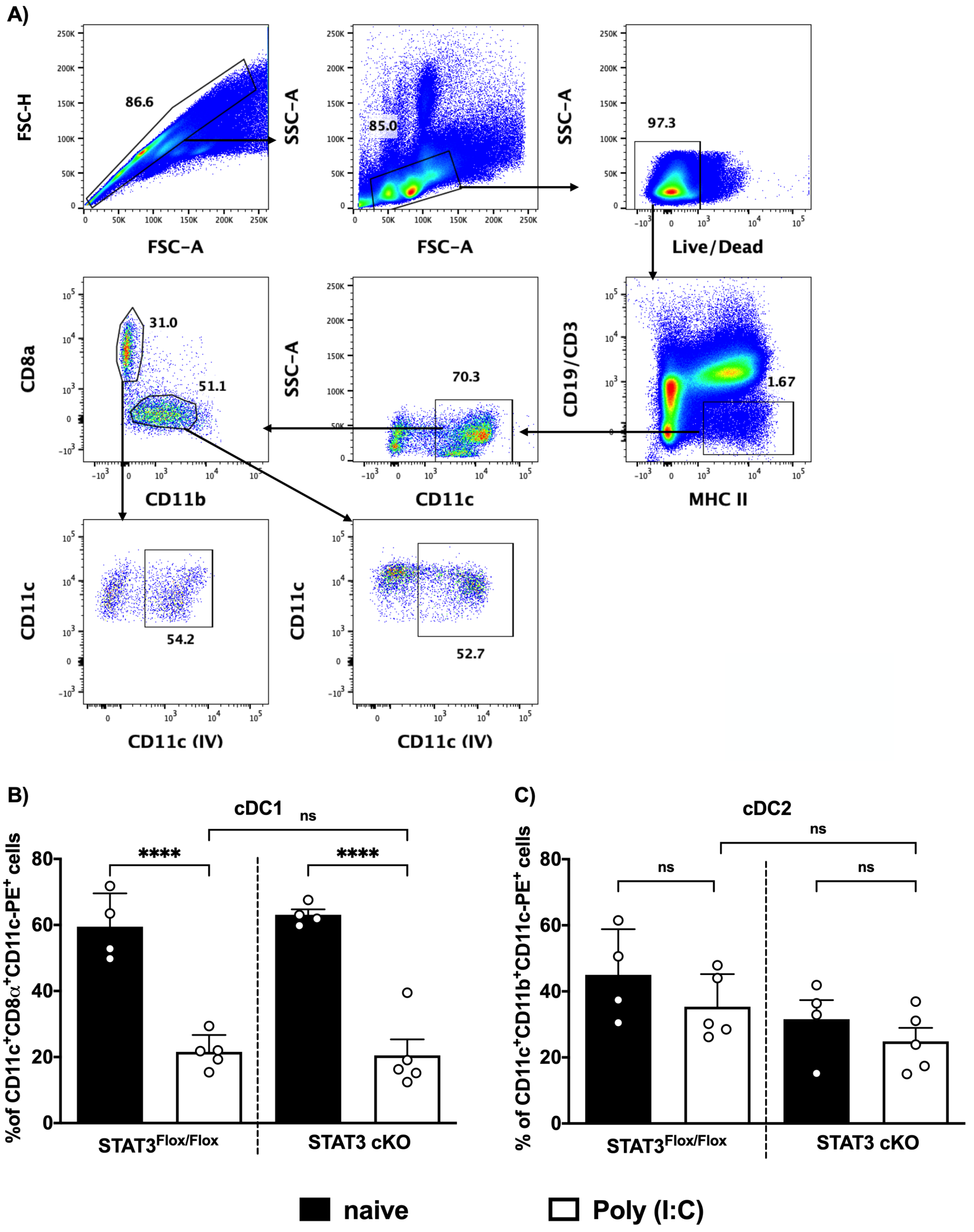

SUPPLEMENTARY FIGURE 11 STAT3 does not impact the intrasplenic migration of cDC1. STAT3$^{Flox/Flox}$ and STAT3 cKO received or not 50 µg of Poly (I:C) intraperitoneally. Six hours later, 1.5 µg of anti-CD11c-PE was administered intravenously. After three minutes, animals were euthanized and splenocytes were obtained and labeled with antibodies conjugated to fluorochromes for

the identification of cDC1 and cDC2 by flow cytometry. **(A)** Gating strategy to analyze cDC1 and cDC2 intrasplenic migration: cells were gated in the singlets, followed in the SSC vs FSC. Then, live cells were selected and CD19/CD3$^-$MHCII$^+$ cells were gated. DC were gated based in CD11c expression. CD11c$^+$ cells were subdivided into CD8α$^+$CD11b$^-$ cDC1s and CD8α$^-$CD11b$^+$ (cDC2s). To evaluate the cDC localization in the red pulp, CD11c(IV)$^+$ cells were gated. **(B)** Frequency of IV$^+$ CD11c cDC1. **(C)** Frequency of IV$^+$ CD11c cDC2. Bars show mean±SD from two experiments pooled together (n=4-5 animals/group). ****p<0.0001 and ns not significant.; one-way ANOVA followed by Tukey's post-test.
